# A generalizable method for the purification and biophysical characterization of bacterial membrane receptors

**DOI:** 10.1002/pro.70825

**Published:** 2026-10-15

**Authors:** Federico Bosetto, Maria Zacharopoulou, Mark Bycroft, Monika Kish, Giovanna Zinzalla, Pamela J. E. Rowling, Stephen H. McLaughlin, Jonathan J. Phillips, Laura S. Itzhaki, Ioanna Mela

**Affiliations:** ^1^ Department of Pharmacology University of Cambridge Cambridge UK; ^2^ Living Systems Institute University of Exeter Exeter UK; ^3^ Department of Biosciences University of Exeter Exeter UK; ^4^ Medical Research Council Laboratory of Molecular Biology Cambridge UK

**Keywords:** biophysical characterization, HasR receptor, HDX‐MS, NMR, protein purification

## Abstract

Membrane‐embedded bacterial receptors are challenging to express and purify in soluble form, yet their isolated domains are essential tools for structural and ligand‐discovery studies. *Pseudomonas aeruginosa* relies on the TonB‐dependent heme receptor HasR for iron acquisition, a process central to its pathogenicity. Here, we report a robust strategy for the recombinant expression, purification, and biophysical characterization of the two soluble HasR domains directly involved in heme uptake: the N‐terminal plug and the Secretin/TonB short N‐terminal domain. Each domain was expressed individually in *Escherichia coli* and purified to homogeneity, adopting well‐folded conformations as confirmed by circular dichroism, NMR spectroscopy, and mass spectrometry. We then engineered a fusion construct containing both domains and systematically evaluated multiple solubilization tags. A GST‐His dual‐affinity strategy enabled efficient purification of the construct, whereas His6‐tag alone resulted in insoluble protein and HLT‐tag fusions suffered from non‐specific proteolysis. Biophysical analyses revealed that the Secretin/TonB short N‐terminal domain remains stably folded within the fusion construct, while the N‐terminal plug domain becomes partially disordered, a finding further supported by hydrogen/deuterium exchange mass spectrometry. Together, these results establish a generalizable workflow for producing soluble receptor domains from membrane proteins and provide validated HasR constructs suitable for downstream ligand‐screening applications, including aptamer and nanobody discovery.

## 1 INTRODUCTION

*Pseudomonas aeruginosa* is a Gram‐negative bacterium, known for its remarkable capability to survive in diverse environments, as it can thrive on minimal nutrients and can tolerate a broad range of environmental conditions (Farag et al., 2024; Lister et al., 2009). This adaptability allows it to persist in both community settings and, notably, in the challenging environment of healthcare facilities, where it appears as an opportunistic pathogen causing infection and mortality in cystic fibrosis patients and immunocompromised individuals (Farag et al., 2024; Lister et al., 2009; Pang et al., 2019). *Pseudomonas aeruginosa* is resistant to several classes of antibiotics, including aminoglycosides, quinolones, and β‐lactams, due to a combination of different factors including intrinsic, acquired, and adaptive resistance mechanisms (Farag et al., 2024; Krūmiņa et al., 2026; Pang et al., 2019). Antibiotic resistance shown by *P. aeruginosa* is due to intrinsic and acquired traits, such as low permeability of the outer membrane, efflux pumps that actively transport antibiotics out of the cell, and the production of β‐lactamases and aminoglycoside‐modifying enzymes that impair antibiotic activity. Furthermore, *P. aeruginosa* can form biofilms, which act as a physical barrier to antibiotic penetration into bacterial cells (Farag et al., 2024; Krūmiņa et al., 2026; Pang et al., 2019). *Pseudomonas aeruginosa* belongs to the group of ESKAPE pathogens, represented by the six most multidrug‐resistant bacteria and for which the development of new treatments is necessary, including: *Enterococcus faecium*, *Staphylococcus aureus*, *Klebsiella pneumoniae*, *Acinetobacter baumannii*, *P. aeruginosa*, and *Enterobacter* spp (Miller & Arias, 2024).

Iron is essential for bacterial growth (Dent & Wilks, 2020; Smith & Wilks, 2015), and plays a significant role in various cellular functions, such as energy production, DNA replication, electron transport, and virulence (Andrews et al., 2003). In vertebrates, iron is mainly coordinated within a porphyrin ring structure named heme (Becker & Skaar, 2014; Marchetti et al., 2020; Sánchez‐Jiménez et al., 2023) which is responsible for the transport and storage of oxygen in proteins such as hemoglobin and myoglobin (Becker & Skaar, 2014; Marchetti et al., 2020). Sequestering heme within proteins is a well‐documented strategy adopted by vertebrates to prevent bacterial infections; however, many pathogens have developed mechanisms to utilize heme as a source of iron (Becker & Skaar, 2014; Marchetti et al., 2020). In *P. aeruginosa*, three main heme uptake systems are known: the heme assimilation system (*Has*) (Dent & Wilks, 2020; Llamas & Sánchez‐Jiménez, 2022; Sánchez‐Jiménez et al., 2023; Smith & Wilks, 2015), the *Pseudomonas* heme uptake (*Phu*) (Dent & Wilks, 2020; Llamas & Sánchez‐Jiménez, 2022; Smith & Wilks, 2015), and the TonB‐dependent *Hxu* system (Otero‐Asman et al., 2019). The Has pathway is unique in its reliance on the secreted hemophore HasA, which captures extracellular heme and delivers it to the outer‐membrane receptor HasR (Figure 1a) (Dent & Wilks, 2020; Llamas & Sánchez‐Jiménez, 2022; Ochsner et al., 2000; Smith & Wilks, 2015) whereas Phu (Dent & Wilks, 2020; Ochsner et al., 2000; Smith & Wilks, 2015) and Hxu (Otero‐Asman et al., 2019) receptors bind free heme directly.

**FIGURE 1:**
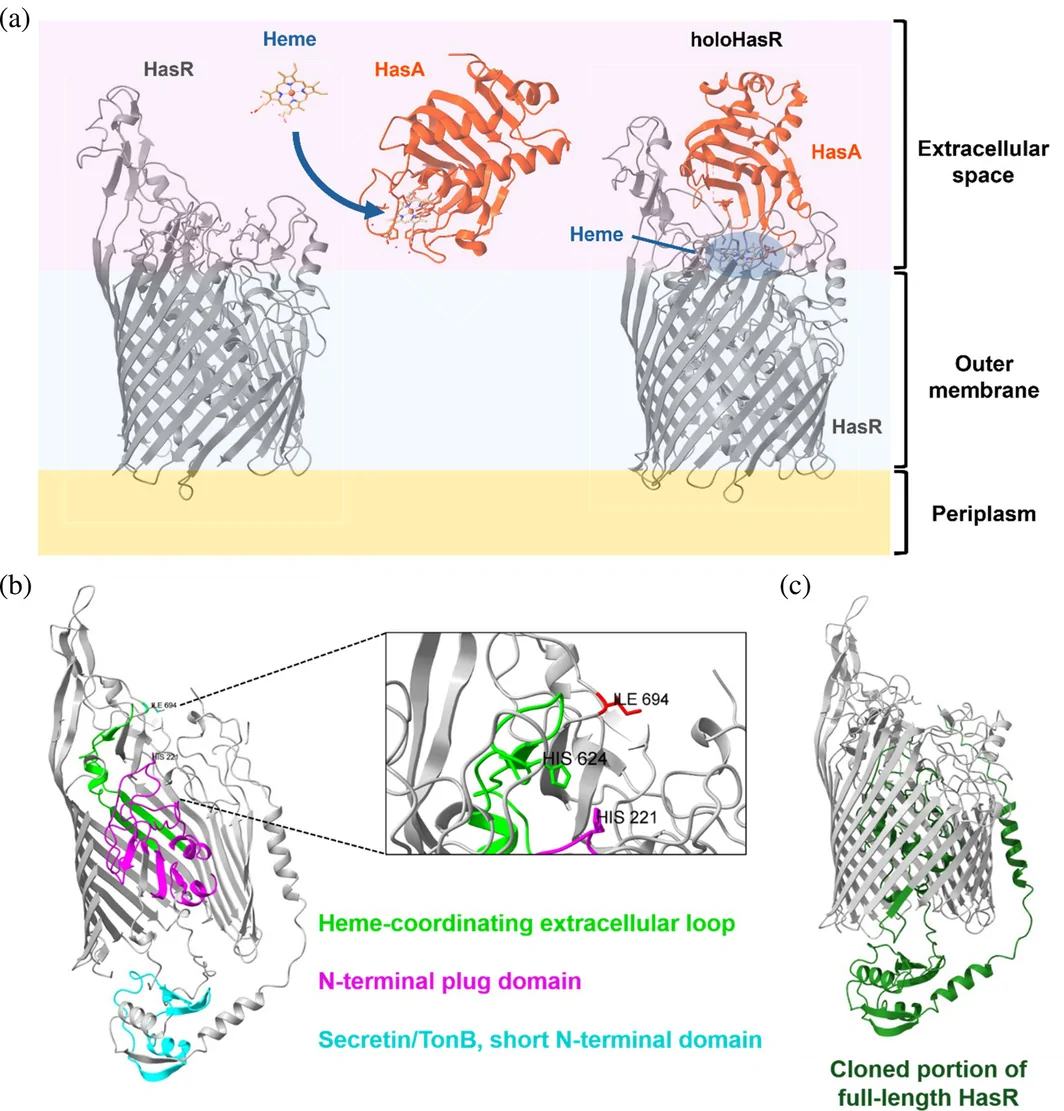
(a) Schematic representation of the heme assimilation system in *Pseudomonas aeruginosa*, constituted by the receptor HasR (in gray) and the hemophore HasA (in orange) which binds the heme (blue arrow), and together form the holoHasR. The image was adapted from PDB file 3CSL (Krieg et al., 2009) in ChimeraX 1.8. (b) Illustration of the three HasR domains: Secretin/TonB short N‐terminal (in cyan), N‐terminal plug (in purple), and heme‐coordinating extracellular loop (in light green); where the last two are directly involved in the heme coordination binding thanks to His‐221 and His‐624 residues. The beta barrel structure is partially concealed to allow for visualization of the domains. (c) Highlight of the cloned portion of HasR including both N‐terminal plug and Secretin/TonB short N‐terminal domains (in dark green). The construct model was generated with AlphaFold 3 using the AlphaFold Server (accessed March 5, 2025) and rendered in ChimeraX 1.8 (Meng et al., 2023).

HasR (Figure 1a) is a TonB‐dependent transporter composed of three domains: a short N‐terminal Secretin/TonB domain, an N‐terminal plug domain, and an extracellular heme‐coordinating loop (Figure 1b). The plug and extracellular loop contain key residues—His‐221, His‐624, and Ile‐694—that mediate heme transfer from holo‐HasA, regulate transport, and prevent heme back‐transfer to HasA (Dent & Wilks, 2020). The importance of iron acquisition is central to bacterial survival, the soluble domains of HasR represent attractive targets for therapeutic ligand discovery. Currently, therapeutic strategies targeting iron metabolism rely on iron chelators, hemolysins inhibitors, the use of gallium as iron analog, and antibiotic‐modified siderophores (Hoffmann et al., 2025). Therefore, the ability to specifically target the functional soluble domains of receptors, such as HasR, could represent an important and alternative approach to combating antibiotic‐resistant bacteria.

No experimental structure of *P. aeruginosa* HasR has been reported; however, the homologous receptor from *S. marcescens* has been characterized in detail: the full‐length receptor has been crystallized in complex with its hemophore HasA, both with and without bound heme (PDB 3CSL and 3CSN) (Krieg et al., 2009), and the isolated periplasmic signaling domain has been determined by solution NMR (PDB 2M5J) (Malki et al., 2014).

Although full‐length membrane receptors are often difficult to express in soluble form due to hydrophobic transmembrane regions (Hardy et al., 2016), their isolated extracellular and periplasmic domains can frequently be produced recombinantly and purified under native conditions (Alberts et al., 2002; Carpenter et al., 2008). Such soluble domains are valuable tools for structural studies and for screening ligands—including aptamers or nanobodies—that may disrupt receptor function. However, expressing isolated domains of membrane proteins presents challenges: domain boundaries must be carefully selected (de Marco, 2025; Tungekar et al., 2021), solubility and folding can be compromised outside the native membrane environment, and purification often requires tailored strategies or solubilization tags.

A carefully designed purification strategy is critical for ensuring yield, purity, and functionality. Affinity tags such as His, Glutathione S‐Transferase (GST), Small Ubiquitin‐like Modifier (SUMO), or Maltose Binding Protein (MBP) can enhance solubility and folding, especially when fused to the N‐terminus of the protein of interest (Dyson et al., 2004; Jia & Jeon, 2016). Hexa‐histidine (His6) tags are the most widely used of these because they are small, are only rarely perturbing to folding, and can be placed at either terminus. They permit capture by immobilized metal‐affinity chromatography (IMAC), in which the imidazole side chains of the tag coordinate divalent transition‐metal ions, typically Ni^2+^ that are immobilized on a chelating resin; host proteins that lack a comparable run of surface histidines are washed away, and the tagged protein is subsequently recovered by competitive elution with imidazole. Chromatographic techniques—including ion exchange (IEX), hydrophobic interaction (HIC), and size‐exclusion chromatography (SEC)—are frequently required to achieve high purity. SEC is especially valuable not only for removing impurities and aggregates but also for analyzing the size and oligomeric state of the protein of interest (monomer/oligomers) (Hong et al., 2012).

Expressing and purifying only the soluble domain of the protein of interest allows one to avoid the use of detergents entirely (Krishnarjuna & Ramamoorthy, 2022) while preserving structural integrity. However, if residual hydrophobic patches remain, mild detergents are sometimes necessary. Strategies to increase overall protein yield include lower induction temperatures, reduced IPTG levels, and initiating induction during late log phase (Galloway et al., 2003; Sørensen & Mortensen, 2005), matching codon usage to the host organism, or co‐expressing chaperones (de Marco et al., 2007). Expression can also be improved by auto‐induction, in which cultures are grown in a defined medium containing glucose and lactose so that induction occurs automatically as glucose is exhausted in late log phase; this removes the need to monitor OD600 and to add IPTG at a defined point, allows unattended growth to high cell density, and has proved particularly effective for targets that express poorly under conventional IPTG induction (Studier, 2005). Once purified, biophysical methods such as circular dichroism (CD) (Greenfield, 2006), nanoDSF (Gao et al., 2020), NMR (Cavanagh et al., 2007; Rehm et al., 2002), and ligand‐binding assays are essential to confirm correct folding and functionality.

Here we report a rapid and robust protocol for the expression and purification of the soluble domains of HasR: the N‐terminal plug, the Secretin/TonB short N‐terminal domain, and a fusion construct containing both (Figure 1b,c). The two domains were first expressed and characterized individually, and subsequently as a single contiguous construct, reflecting their proximity within the amino‐acid sequence of HasR (Figure 2a,b). The heme‐coordinating extracellular loop, also involved in heme binding, was not included as it is separated from these domains by the membrane‐embedded β‐barrel and is not accessible to targeting molecules. We evaluate multiple fusion‐tag strategies, optimize expression and solubility, and establish a purification workflow combining affinity chromatography, SEC, and ion‐exchange polishing. Finally, we characterize the folding and stability of the purified domains using CD, nanoDSF, NMR, and HDX‐MS (Konermann et al., 2011; Masson et al., 2019). Our work provides validated HasR constructs suitable for downstream ligand‐discovery applications and offers a generalisable strategy for producing soluble domains of membrane‐associated bacterial receptors.

**FIGURE 2:**
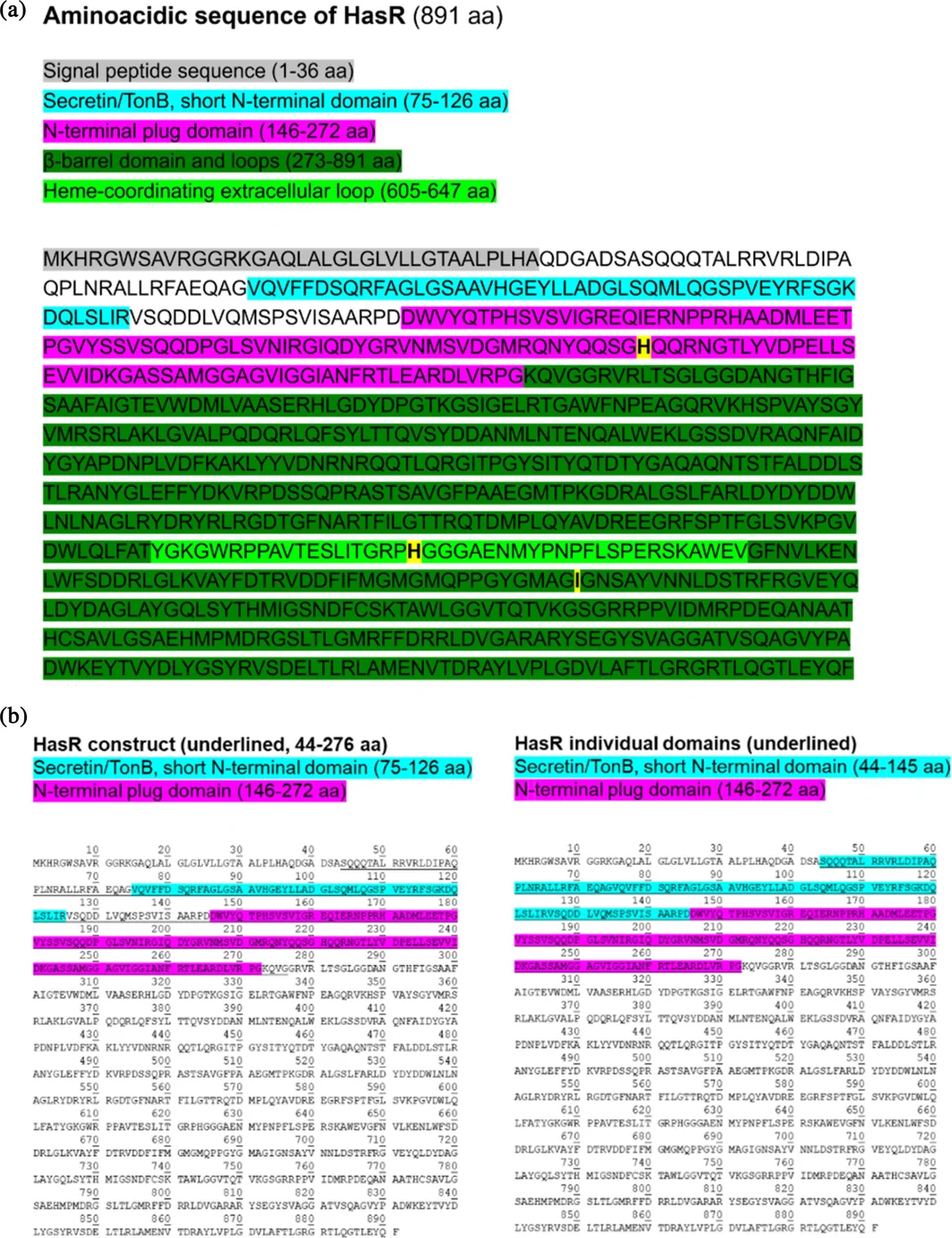
HasR aminoacidic sequence and domains. (a) Schematic of the aminoacidic sequence of HasR, highlighting the different domains: Signal peptide (in gray), Secretin/Ton B short N‐terminal (in cyan), N‐terminal plug (in purple), β‐barrel and loops (in dark green), and heme‐coordinating extracellular loop (in light green). Heme binding residues are highlighted in yellow: H221, H624 and I694. (b) Aminoacidic sequences of HasR construct and HasR individual domains used in this work.

## 2 RESULTS

### 2.1 Recombinant individual HasR domains can be purified and are soluble

To determine whether the soluble regions of HasR could be produced recombinantly, we first expressed the N‐terminal plug and Secretin/TonB short N‐terminal domains individually (Figures 1b,c and 2a,b). Domain boundaries were defined according to the InterPro classification system (Blum et al., 2025); specifically, IPR037066 for the N‐terminal plug domain and IPR011662 for the Secretin/TonB short N‐terminal domain. Furthermore, the latter one was extended at both N‐ and C‐termini to include the linker sequences located downstream of the signal peptide and upstream of the N‐terminal plug domain, respectively (Figure 2a,b). Both proteins were soluble in *Escherichia coli* lysates, although the Secretin/TonB domain expressed at substantially higher levels.

The N‐terminal plug domain was purified through a three‐step purification process due to the presence of high amount of host‐derived contaminants. The first step was performed via nickel affinity chromatography resulting in a well‐defined protein band localized between 23 and 18 kDa, but with protein contaminants at lower and higher molecular weight (Figure 3a,b). The second purification step of anion‐exchange chromatography (ANEX) removed a portion of host‐derived contaminants (Figure 3c,d) and the third and final purification by SEC produced a single major peak corresponding to the plug domain (Figure 3e,f). ESI mass spectrometry confirmed a dominant species at 16.9 kDa, consistent with the predicted mass of 15.8 kDa (Figure S1a). The total yield of the N‐terminal plug domain resulted in ~0.075 mg/L (0.45 mg from 6 L).

**FIGURE 3:**
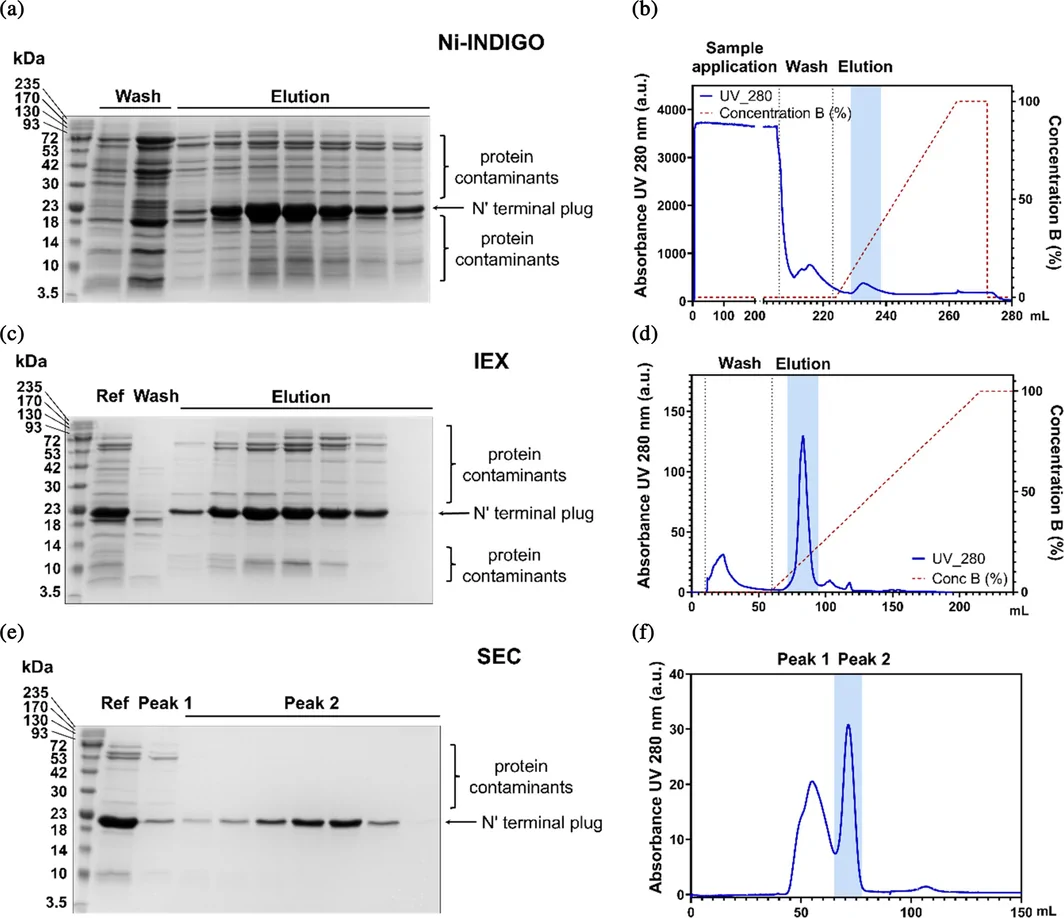
N‐terminal plug domain purification. The protein is initially purified by affinity chromatography using Ni‐INDIGO column as reported by (a) the SDS‐PAGE and (b) the chromatogram from the AKTA. N‐terminal plug protein band is denoted by arrow between 23 and 18 kDa, protein contaminants are present both at higher and lower molecular weight. The chromatogram reports Sample application, Wash and Elution phases; elution peak is highlighted by blue shadow. The second purification step was carried out via IEX using a MonoQ column, again (c) SDS‐PAGE and (d) the chromatogram from the AKTA are shown. N‐terminal plug protein band is denoted by arrow between 23 and 18 kDa, some protein contaminants are still present both at higher and lower molecular weight. The chromatogram reports Wash and Elution phases; elution peak from N‐terminal plug is highlighted by blue shadow. The final step was performed by SEC using a Superdex 75 10/300 GL column, which separated the protein of interest from residual contaminants, as seen in (e) the SDS‐PAGE and (f) AKTA chromatogram. N‐terminal plug protein band is denoted by arrow between 23 and 18 kDa (Peak 2), while a few protein contaminants are still present at higher molecular weight (Peak 1). The chromatogram reports Peak 1 and Peak 2; elution peak from N‐terminal plug is highlighted by blue shadow (Peak 2).

The Secretin/TonB short N‐terminal domain was initially purified by nickel affinity chromatography, yielding a clear, defined protein band at approximately 10 kDa (Figure 4a,b). SEC removed remaining impurities, yielding a homogeneous protein. (Figure 4c,d). The purity and correct molecular weight of the purified protein of 13.1 kDa was confirmed via ESI mass spectrometry (Figure S1b). The total yield of the Secretin/TonB short N‐terminal domain resulted in ~12.3 mg/L (12.3 mg from 1 L).

**FIGURE 4:**
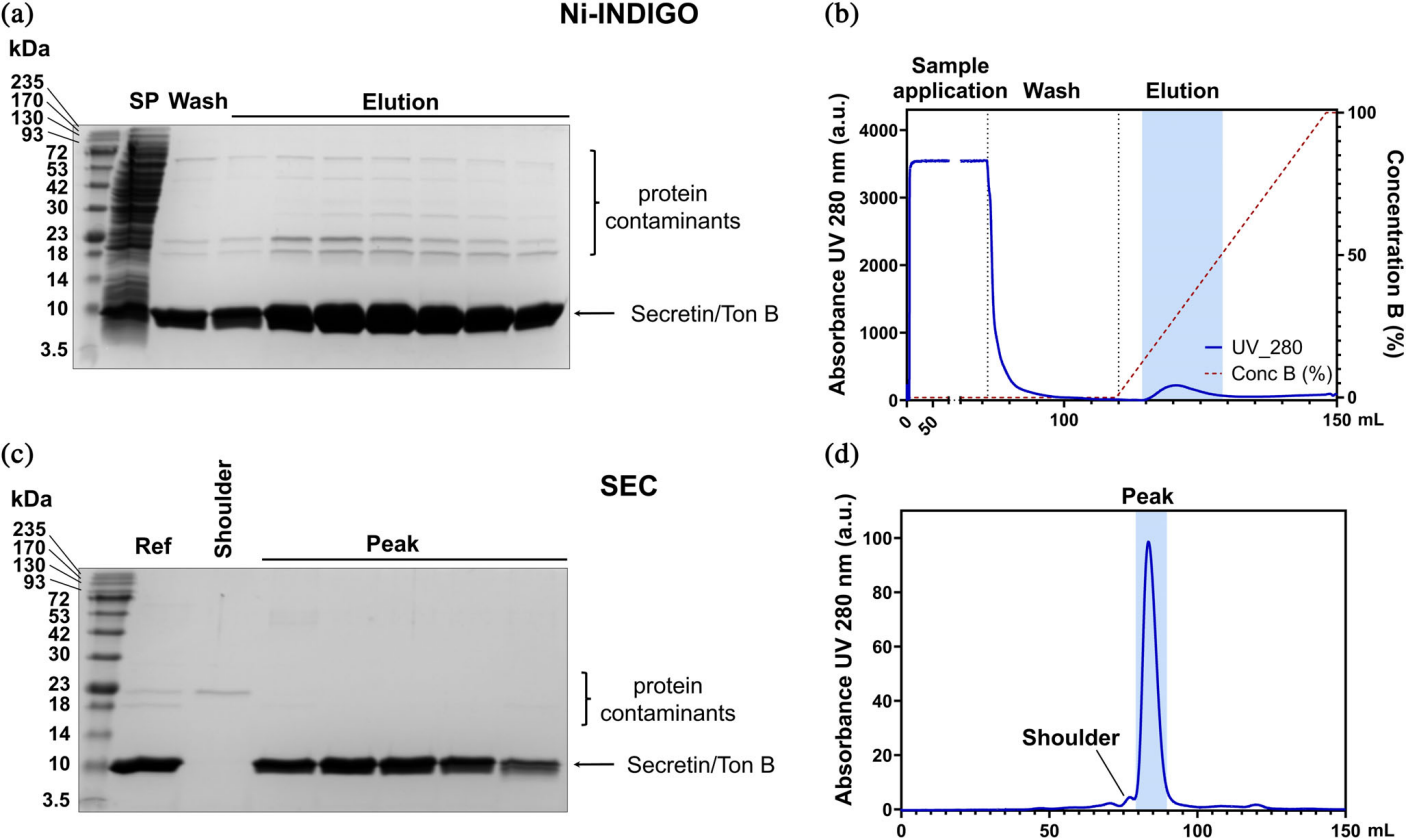
Secretin/TonB short N‐terminal domain purification. The protein is initially purified by affinity chromatography using Ni‐INDIGO column as reported by (a) the SDS‐PAGE and (b) the chromatogram from the AKTA. Secretin/TonB short N‐terminal protein band is denoted by arrow around 10 kDa, protein contaminants are present at higher molecular weight. The chromatogram reports Sample application, Wash and Elution phases; elution peak is highlighted by blue shadow. The second purification step was carried out via SEC using a Superdex 75 10/300 GL column (Cytiva, UK) to isolate the protein of interest, again (c) SDS‐PAGE and (d) the chromatogram from the AKTA are shown. Secretin/TonB short N‐terminal protein band is denoted by arrow around 10 kDa (Peak), and complete removal of protein contaminants is visible (Shoulder). The chromatogram reports Peak and Shoulder; elution peak from Secretin/TonB short N‐terminal protein is highlighted by blue shadow (Peak). SP, soluble protein fraction.

### 2.2 Individual HasR domains adopt well‐folded conformations

CD analysis revealed distinct secondary‐structure profiles for the two domains. Beta Structure Selection (BeStSel) server was used to determine the protein secondary structure and fold recognition from CD spectrum, giving an indication of α‐helix and β‐sheets content (Micsonai et al., 2018). The N‐terminal plug domain exhibited a mixed architecture dominated by antiparallel β‐strands (37%), with 2% distorted α‐helices, 18% β‐turns and 44% others (disordered) (Figure 5a,c), while the Secretin/TonB short N‐terminal domain was constituted by 27% α‐helices, 17% β‐strands, 14% β‐turns and 42% others (disordered) (Figure 5b,d). These observations were further supported by 1D NMR spectra. The 1D NMR spectrum of the N‐terminal plug is well dispersed, characteristic of a fully folded protein (e.g., peaks with low chemical shift values, between −0.5 and 0.5 ppm are resolved) (Figure 6a). Interestingly, the resolved NH signals that are present only when the plug domain is folded are typical of β‐sheets. Similarly, the 1D NMR spectrum of the Secretin/TonB short N‐terminal domain showed well‐dispersed spectra, characteristic of a well‐folded structure (Figure 6b). These observations were further substantiated by a 2D NOESY experiment, where widely dispersive peaks indicated long‐range, through‐space contacts that are only present in a folded structure (Figure S2).

**FIGURE 5:**
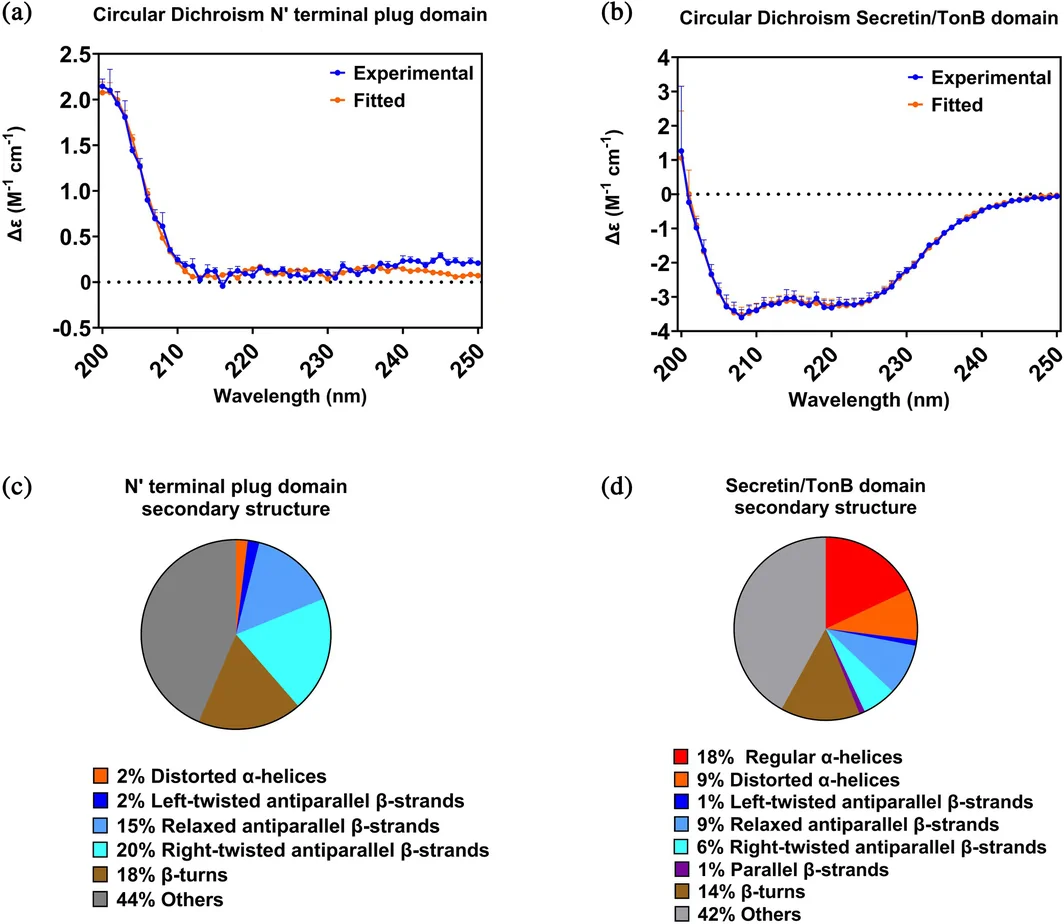
Circular Dichroism spectra of the N‐terminal plug (a) and Secretin/TonB short N‐terminal domains (b) were measured in three technical replicates at 25°C between 200 and 250 nm (in blue), the fitted curves from BeStStel are also reported (in orange). (c) Secondary structure prediction of the N‐terminal plug domain by BeStSel server using the CD values, indicating a composition of approximately 2% distorted α‐helices, 37% antiparallel β‐strands (2% left‐twisted, 15% relaxed, and 20% right‐twisted), 18% β‐turns and 44% others (disordered). (d) Secondary structure prediction of the Secretin/TonB short N‐terminal domain by BeStSel server using the CD values, indicating a composition of approximately 27% α‐helices (18% regular and 9% distorted), 16% antiparallel β‐strands (1% left‐twisted, 9% relaxed and 6% right‐twisted), 1% parallel β‐strands, 14% β‐turns, and 42% others (disordered).

**FIGURE 6:**
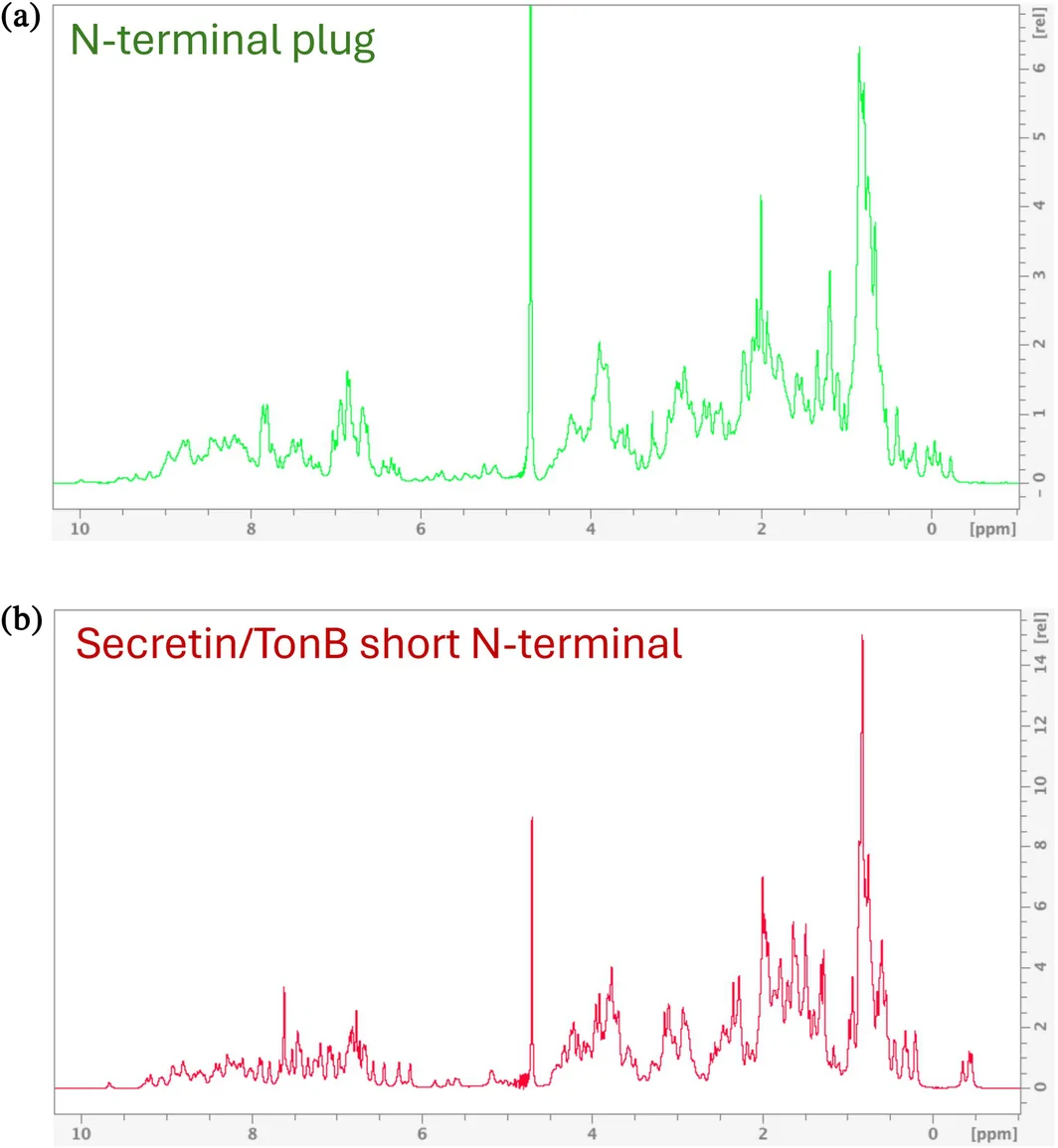
1D NMR spectra of individual N‐terminal plug and Secretin/TonB short N‐terminal domains on an 800 MHz spectrometer. (a) 1D NMR spectrum of individual N‐terminal plug domain. (b) 1D NMR spectrum of individual Secretin/TonB short N‐terminal domain. The dispersion of the chemical shifts in the amide and methyl regions is indicative of these domains adopting a folded structure.

Together, these data demonstrate that both HasR domains are independently soluble and adopt well‐defined tertiary structures.

### 2.3 Recombinant HasR construct requires a solubilization tag fusion

We next engineered a construct containing the N‐terminal plug and the Secretin/TonB short N‐terminal domains in their native sequence order (Figures 1b,c and 2a,b). We evaluated three fusion‐tag strategies to optimize expression and solubility. The HasR dual‐domain construct was fused to either a simple N‐terminal His6 tag, an N‐terminal Histidine–Lipoyl (HLT) solubilization tag (Lebendiker & Danieli, 2014), or an N‐terminal GST tag combined with a C‐terminal His6 tag (Figure S3a–c). Each construct was cloned into pBRE, pHLT, or pGST vectors containing either thrombin or TEV protease cleavage sites to enable removal of the fusion tag after purification.

Since high solubility typically reflects correct folding and greatly simplifies downstream purification, we first assessed the expression behavior of each fusion variant in *E. coli*. Analysis of total, soluble, and insoluble fractions by SDS‐PAGE revealed that the His6‐tagged construct expressed strongly at the expected molecular weight (27.4 kDa) but was almost entirely insoluble (Figure 7a), indicating accumulation in inclusion bodies. Introducing the HLT tag substantially improved solubility, shifting most of the protein to the soluble fraction at ~36.5 kDa (Figure 7b). The GST fusion tag also enhanced solubility (Figure 7c), consistent with GST's well‐established role as a highly soluble carrier protein. These observations indicated that a solubilization tag (HLT or GST) was required for successful production of the HasR dual‐domain construct. The cleavable GST fusion protein confers another advantage besides solubilization: it can be used as a separation step via affinity chromatography with glutathione resin, allowing for a “double‐tag” (GST‐HasR‐His) purification strategy.

**FIGURE 7:**
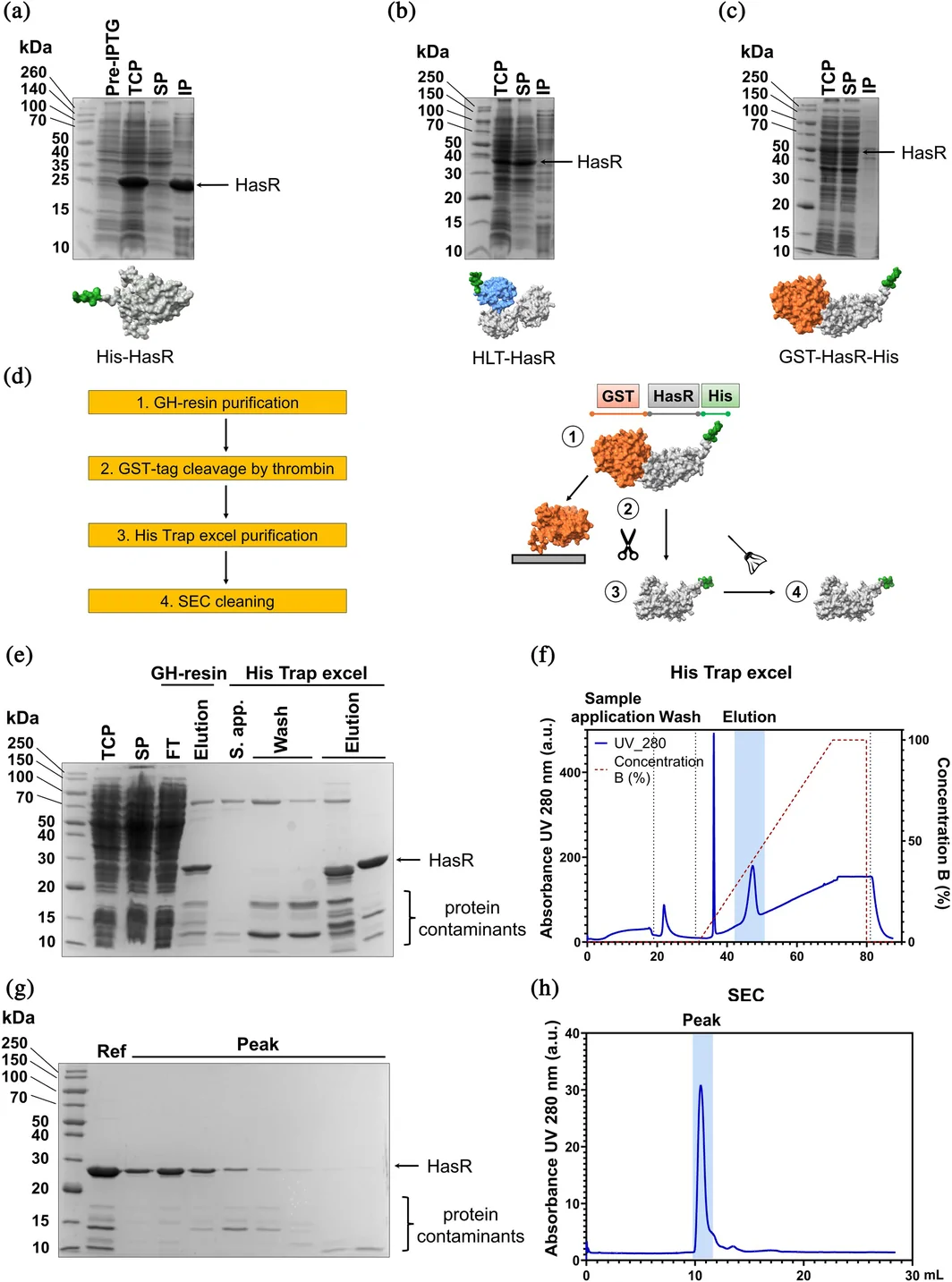
Experimental trials of the HasR construct expression with different fusion tags: (a) N‐terminal HexaHistidine, (b) N‐terminal HLT and (c) N‐terminal GST with C‐terminal HexaHistidine. HasR construct is denoted by the arrow. TCP, total cell protein lysate; SP, soluble protein fraction; IP, insoluble protein fraction. HasR‐His6 is primarily expressed in the insoluble fraction (a), while the addition of the HLT tag (b) or GST (c) drives the expression to the soluble part. GST‐HasR‐His6 purification and characterization. (d) Flow chart of the GST/His purification process. The protein is first purified by means of GH‐resin, where the GST‐tag is cleaved by Thrombin, and then by affinity chromatography using His Trap excel column, as reported by (e) the SDS‐PAGE and (f) the chromatogram from the AKTA. HasR construct band is denoted by arrow between 30 and 20 kDa, some protein contaminants are present at lower molecular weight. The chromatogram reports Sample application, Wash and Elution phases; elution peak is highlighted by blue shadow. The protein is further purified by SEC, (g) SDS‐PAGE and (h) the chromatogram from the AKTA are shown. HasR construct band is denoted by arrow between 30 and 20 kDa, and a progressive reduction in protein contaminants is clearly visible. The chromatogram reports the elution phase, and the elution peak (Peak) is highlighted by blue shadow.

We next evaluated purification performance. The HLT‐HasR construct was purified by nickel affinity chromatography. HLT‐HasR expressed very well, resulting in a good protein yield, as indicated by the intense protein bands at the elution phase of the SDS‐PAGE gel that represents approximately 70% of the total protein (Figure S4a). Nevertheless, the affinity purification step did not sufficiently purify the protein, as multiple additional proteins from the bacterial lysate were still present in the eluate (Figure S4a). Thrombin cleavage of the HLT tag released full‐length HasR but also generated multiple degradation products (10–15 kDa), and prolonged digestion resulted in complete loss of intact protein (Figure S4b). This pronounced susceptibility to thrombin suggested non‐specific proteolysis and made the HLT strategy unsuitable for further development.

Given these limitations, we focused on the dual‐tag GST‐HasR‐His6 construct, which offered both enhanced solubility and the opportunity for sequential affinity purification. In addition, we evaluated TEV protease as an alternative cleavage strategy, anticipating greater specificity and reduced degradation compared with thrombin (Sun et al., 2012).

### 2.4 Recombinant HasR construct can be efficiently purified via a GST‐tag and His6‐tag affinity chromatography strategy

To purify the dual‐domain GST‐HasR‐His construct, we established a sequential workflow designed to exploit both affinity tags and minimize contaminants. Cultures expressing the construct were grown and induced under conditions favoring soluble expression (Francis & Page, 2010), and the soluble lysate was subjected to a three‐step purification strategy (Figure 7d).

In the first purification step (*Capture—Affinity Separation*), the GST tag enabled capture of the fusion protein on glutathione resin. The total cell lysate was incubated with glutathione resin, the unbound fractions were washed, and the GST fusion tag was cleaved with thrombin, releasing the HasR‐His6 construct, while the GST remained bound to the resin. This step produced a clear HasR band with only minor additional species (Figure 7e).

The eluate was then subjected to a second, nickel affinity chromatography purification step (*Intermediate Purification—Affinity Chromatography*), which removed most remaining contaminants and yielded a dominant HasR band with a small number of lower‐molecular‐weight species attributable to trace impurities or partial degradation (Figure 7e,f). The purity of HasR at this purification step was 79%.

The third, SEC purification step (*Polishing—Gel Filtration*) (Figure 7g,h) removed residual contaminants and produced a single major peak corresponding to the HasR construct. ESI mass spectrometry confirmed the correct molecular weight and high purity (Figure S1c).

These results demonstrate that the dual‐affinity GST‐HasR‐His6 strategy is markedly superior to the single‐tag HLT‐HasR approach, providing higher purity and fewer contaminants. It is a relatively quick and efficient method of purifying proteins of interest to satisfactory purity and can be followed with a final SEC polishing purification step to further elevate the purity. A key optimisation step involved the choice of protease for tag removal. Thrombin cleavage proved unsuitable, as the HasR construct was highly susceptible to non‐specific digestion, resulting in extensive degradation and reduced yield (Figure S4b). In contrast, TEV protease provided specific and efficient cleavage. On‐resin TEV digestion followed by nickel affinity purification (Figure S5a) increased purity to 87% and substantially reduced the presence of 10–20 kDa degradation products (Figure S5b,c). The final yield of purified HasR construct was ~0.5 mg/L (4.19 mg from 9 L culture).

Overall, protein constructs were produced in several independent expression and purification campaigns over the course of this study, at culture scales ranging from 1 to 9 L and using both thrombin and TEV‐based cleavage as described above. Absolute yields varied between batches, as is common for constructs expressed at low levels, but the purification behavior itself was reproducible. In each preparation, the protein eluted at a consistent volume from the SEC column and gave the same band pattern by SDS‐PAGE. Unless stated otherwise, the biophysical measurements reported here were performed on a single representative protein preparation.

### 2.5 HasR construct adopts a partially folded conformation

Having purified the HasR dual‐domain construct to high purity, we next assessed whether it adopted the expected folded architecture. CD spectroscopy revealed a mixed secondary‐structure profile comprising 11% α‐helices, 25% β‐strands, 17% β‐turns, and 49% disordered content (Figure 8a,b). These values closely match AlphaFold3 predictions for the combined domains (25% α‐helix, 25% β‐sheet, ~50% disordered), indicating that the construct contains both structured and intrinsically flexible regions.

**FIGURE 8:**
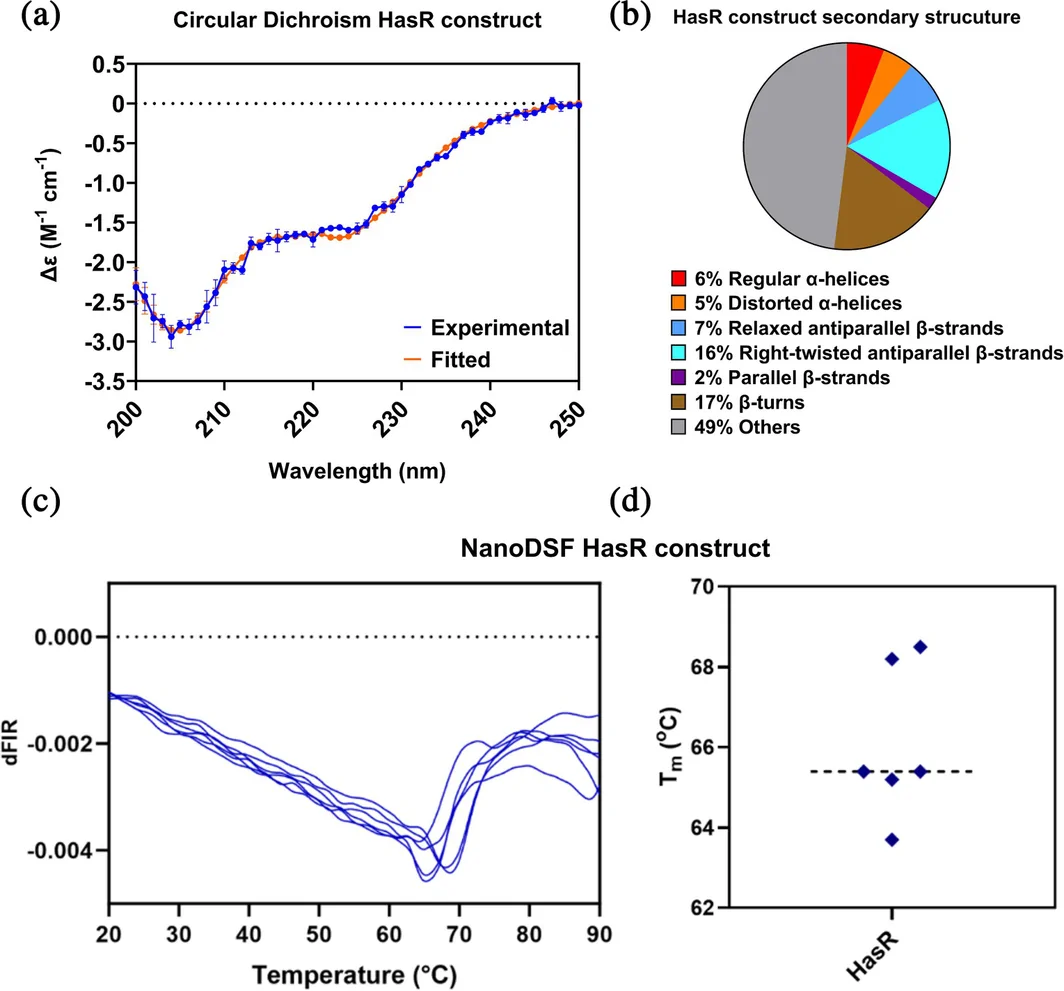
(a) Circular Dichroism spectrum of HasR construct measured in three technical replicates at 25°C between 200 and 250 nm (in blue), the fitted curve is also reported (in orange). (b) Secondary structure prediction by BeStSel server using the CD values, indicating a composition of approximately 11% of α‐helices (6% regular and 5% distorted), 23% of antiparallel β‐strands (7% relaxed and 16% right‐twisted), 2% parallel β‐strands, 17% β‐turns, and 49% others (disordered) of HasR‐His. (c) Unfolding of purified HasR construct determined by Nano Differential Scanning Fluorimetry (NanoDSF). The data shown are the first derivative of the ratio of fluorescence intensity read at 350 nm over that at 330 nm (dFIR [350 nm/330 nm]). The global minimum corresponds to the melting temperature of the protein, T_m_. (d) Extracted *T*_*m*_ values from the NanoDSF traces indicate that the protein is folded and that it has moderate thermal stability (66 ± 1.7°C), typical for many soluble, functional proteins. The NanoDSF experiments were performed with a Prometheus NanoDSF instrument (NanoTemper Technologies). Thermal denaturation was performed from 20 to 90°C with a 1°C/min rate.

Thermal stability was evaluated using nanoDSF (Figure 8c,d). Monitoring intrinsic tryptophan and tyrosine fluorescence during heating from 20 to 90°C yielded a melting temperature of 66 ± 1.7°C. This Tm falls within the range typical for soluble bacterial proteins (53–73°C), suggesting that the folded regions of the construct are moderately stable (Pucci & Rooman, 2016).

To probe tertiary structure, we collected 1D NMR spectra of the purified protein (Figure 9a). The spectrum displayed well‐dispersed resonances characteristic of folded domains, alongside numerous amide signals in the random‐coil region, consistent with the substantial disorder observed by CD. Together, these data indicate that the HasR construct contains a folded core with additional flexible regions.

**FIGURE 9:**
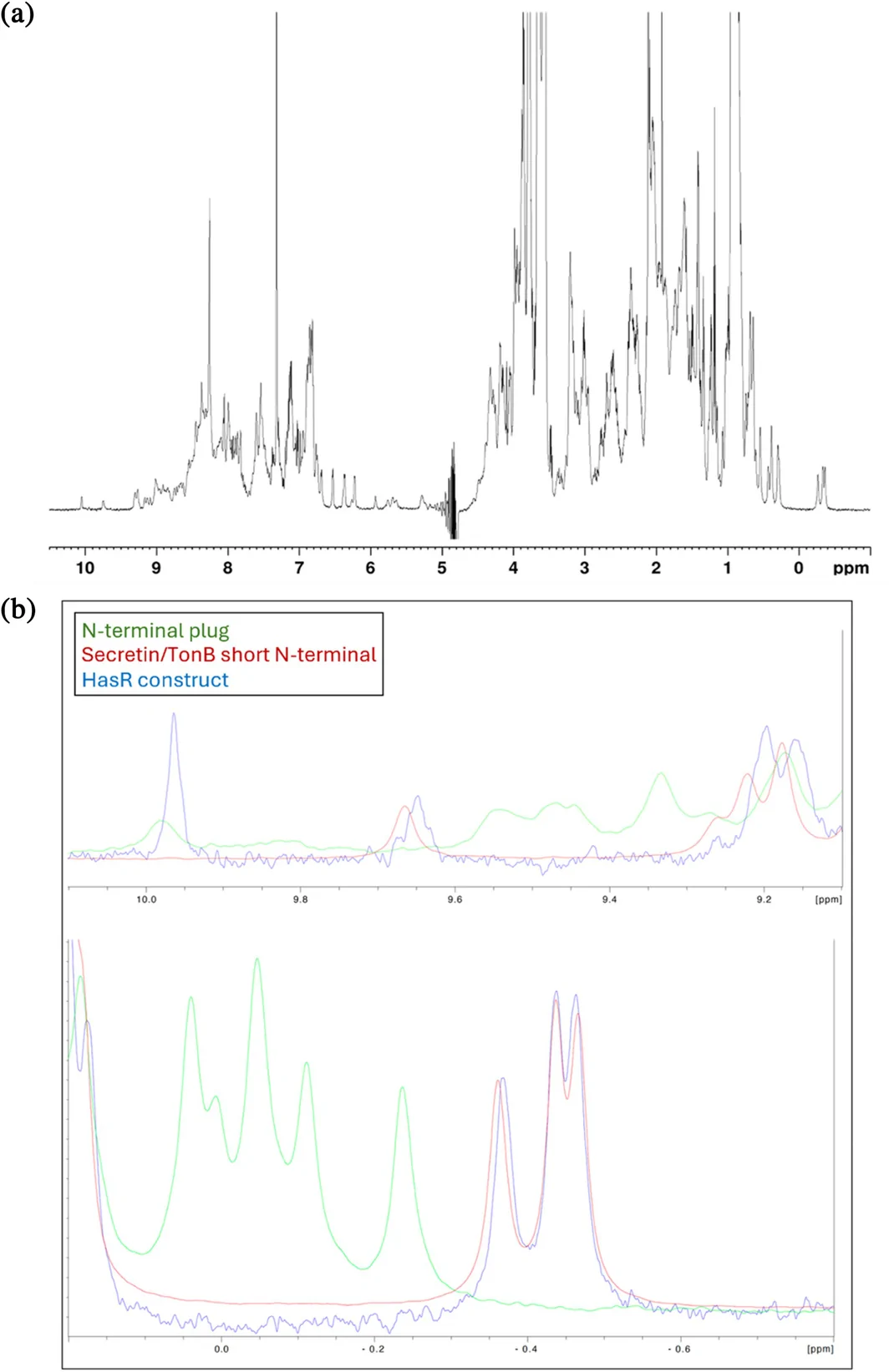
NMR spectra of HasR construct and comparison with NMR spectra of HasR individual domains on an 800 MHz spectrometer. (a) 1D 1H spectrum of the HasR construct. (b) 1D 1H spectra overlay of the folded and unfolded states of a protein as comparison to illustrate that the HasR construct is folded. (c) Superimposition of the 1D spectra of the methyl (above) and amide regions (below) of the three constructs (N‐terminal plug in green, Secretin/TonB short N‐terminal in red and HasR construct in blue), illustrating that the signals from the folded N‐terminal plug domain are not present in the HasR construct.

To determine whether both domains retained their native folds within the fusion construct, we compared NMR spectra of the individual domains with that of the combined protein (Figure 9b). The resonances corresponding to the fully folded N‐terminal plug domain (green trace) are not present in the spectrum of the HasR construct (blue trace), indicating the plug domain does not adopt the same well‐packed β‐sheet conformation when expressed together with the Secretin/TonB short N‐terminal domain. In contrast, the resonances corresponding to the Secretin/TonB domain (red trace) were preserved, indicating that this domain remains stably folded in both contexts.

Finally, to obtain residue‐level insight into structural stability, we performed Hydrogen/Deuterium Exchange Mass Spectrometry (HDX‐MS). The protein was digested efficiently, yielding a coverage map of 88 peptides with 100% coverage and 5.02 average redundancy, as well as areas of very high redundancy (>10 peptides) (Figure S6). H/D labeling across five timepoints spanning milliseconds to minutes (300 ms, 500 ms, 1 s, 30 s, 5 min) allows for capturing fast and slower exchange kinetics (Figure S7). The exchange data for each peptide were averaged and represented as a heat map across the protein sequence, where yellow color corresponds to lower deuterium uptake (stable/folded structure), and red color corresponds to higher deuterium uptake (dynamic/unfolded structure).

HDX showed that the Secretin/TonB short N‐terminal domain is a fully folded and stable region, as the deuterium labeling was low (<40%, yellow), indicating a natively folded structure (Figure 10a). The N‐terminal plug domain showed increased deuterium uptake, reaching 62%, which corresponds well with the loop regions (amino acids 160–180) that are expected to be very flexible. Importantly, intermediate deuterium uptake was observed in the expected β‐strand regions (amino acids 130–150, 200–220), indicating a weakly stable structure, rather than complete disorder. Mapping HDX values onto the AlphaFold3 model of the construct (Figure 10b) and comparing them with annotated domain boundaries (Figure 10c) confirmed this domain‐specific behavior.

**FIGURE 10:**
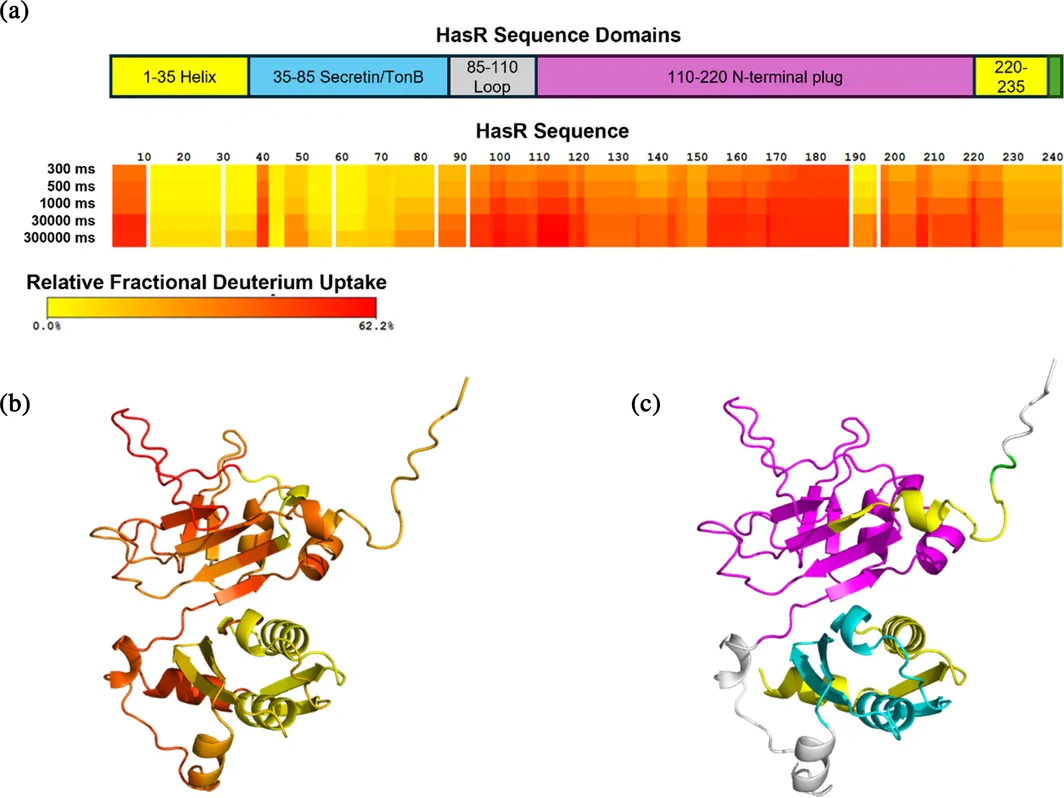
HDX‐MS of HasR construct indicates folded structure. (a) Deuterium Uptake Heatmap across the sequence of the HasR construct. Areas in yellow indicate lower deuterium uptake, corresponding to folded and stabler areas of the protein. Areas in red indicate higher deuterium uptake, corresponding to more dynamic or unfolded areas of the sequence (e.g., loops). The protein domains (Helix, Secretin/TonB, N‐terminal plug) are also highlighted. (b) Superimposition of the deuterium uptake data on the HasR construct 3D structure. (c) Highlighted sequence domains on the HasR construct 3D structure (corresponding to a), to aid comparison. The construct model was generated with AlphaFold 3 using the AlphaFold Server (accessed March 5, 2025) and rendered in PyMOL 3.0 (Schrödinger, 2021).

Overall, the biophysical data demonstrate that the Secretin/TonB short N‐terminal domain remains stably folded within the fusion construct, whereas the N‐terminal plug domain becomes only weakly structured, with extensive flexibility in loop regions. These findings align with the NMR and CD results and reveal that domain context strongly influences the folding behavior of HasR's soluble regions.

## 3 DISCUSSION

In this study, we established a robust workflow for expressing, purifying, and characterizing soluble domains from membrane‐associated bacterial receptors, using the soluble N‐terminal domains of the TonB‐dependent heme receptor HasR from *P. aeruginosa* as a model system. By combining systematic tag screening, dual‐affinity purification, and orthogonal biophysical analyses, we generated high‐quality constructs suitable for downstream structural and ligand‐discovery applications. Our findings highlight both the opportunities and the challenges inherent in working with isolated domains of membrane‐embedded receptors.

A central outcome of our work is the identification of an effective solubilization and purification strategy for the HasR dual‐domain construct. Initial trials showed that simple His6‐tag fusion was insufficient, yielding predominantly insoluble protein. Introducing engineered solubility tags significantly improved expression behavior, with both HLT and GST fusions driving the construct into the soluble fraction. Among these, the GST‐HasR‐His dual‐tag design proved most advantageous, providing high solubility, compatibility with sequential affinity purification, and robust performance across multiple purification steps.

The dual‐affinity approach (GST capture followed by His6‐tag enrichment) offered several methodological benefits. First, it enabled efficient removal of host proteins early in the process, reducing the burden on downstream chromatographic steps. Second, it allowed purification under mild, native conditions without the need for detergents, which is particularly valuable for membrane‐associated domains that may be sensitive to denaturation. Third, the workflow was readily adaptable, permitting optimisation of protease cleavage conditions and integration of SEC polishing to achieve high purity.

The final purification strategy—GST affinity capture, TEV cleavage, His‐tag enrichment, and SEC polishing—yielded high‐purity protein suitable for downstream biophysical analysis. Importantly, this workflow is modular and broadly applicable. The combination of solubilization tags, dual‐affinity steps, and orthogonal polishing can be readily adapted to other membrane‐associated receptors or challenging bacterial proteins. The approach is also compatible with large‐scale production, as demonstrated by the reproducible yields obtained across multiple liters of culture.

Beyond purification, the study establishes a methodological framework for validating the structural integrity of soluble receptor domains. Here, on the structure prediction front, AlphaFold was used to visualize the domain organization of HasR, to confirm that the chosen construct termini fell in flexible regions rather than within secondary‐structure elements, and to provide the structural framework onto which the HDX‐MS data were mapped. The structure prediction field is advancing rapidly and in directions that are directly relevant to receptors of this kind: AlphaFold3 and related all‐atom methods extend prediction to complexes with ligands and cofactors, which for a heme‐binding receptor raises the prospect of modeling the holo state rather than the apo protein alone. We would expect future implementations of the workflow described here to begin with a prediction‐guided design stage in which several boundary variants are modeled with more than one method and ranked by confidence (Abramson et al., 2024; Jumper et al., 2021), and followed by their expression, purification, and biophysical characterization. CD spectroscopy, nanoDSF, NMR, and HDX‐MS together provide a comprehensive toolkit for assessing folding, stability, and suitability for downstream applications such as ligand screening. The integration of these techniques ensures that purified constructs are not only homogeneous but also structurally competent for functional studies. Importantly, the folding behavior of the two HasR domains was consistent with expectations from individual expression trials: the Secretin/TonB short N‐terminal domain remained well folded both as an isolated protein and within the dual‐domain construct, demonstrating that its structural integrity is preserved across expression formats. In contrast, the N‐terminal plug domain displayed conformation‐dependent behavior, adopting a stable fold when expressed alone but exhibiting weaker folding within the combined construct. These observations suggest that flexible loop regions within the plug domain exert a greater influence on its folding when the domain is expressed in the context of the larger construct. Together, these results highlight the value of incorporating orthogonal biophysical methods into purification workflows to ensure that recombinant receptor domains retain the structural features required for downstream biochemical and ligand‐discovery studies.

Bringing these observations together, we outline below the sequence of steps we would recommend when approaching a new membrane‐associated receptor domain:

### 3.1 Recommended workflow

1. Define the construct before cloning. Combine domain annotation and an alignment against homologs of known structure and inspect a predicted model such as AlphaFold to confirm that the proposed termini lie in flexible segments and not in structural domains.
2. Screen tagged protein variants. Include the His6 tag alone as a baseline and at least two solubility‐enhancing fusions of differing character; we compared His6, HLT, and GST. Judge the outcome on the distribution between soluble and insoluble fractions by SDS‐PAGE of total, soluble, and insoluble material.
3. Prefer a dual‐tag design. An N‐terminal solubilizing and affinity tag combined with a small C‐terminal tag permits two orthogonal capture steps. The GST–HasR–His6 construct gave markedly better purity than the single‐tag alternative.
4. Screen proteases for tag removal, preferably as a time course. Susceptibility to non‐specific proteolysis is target‐specific and is not predictable: thrombin, although it released the target, digested HasR extensively, whereas TEV protease cleaved cleanly.
5. Polish. Proceed by SEC, adding ion exchange where host contaminants persist, and confirm the identity and integrity of the product by ESI‐MS.
6. Validate folding. We would propose the following: CD for secondary‐structure content, 1D ^1^H NMR for evidence of folding, and nanoDSF for thermal stability. HDX‐MS can be added where residue‐level information about which regions are structured is needed. Critically, compare the domain in isolation with the domain in its final construct context, since the two may differ.
7. Scale up. Proceed to large‐scale production and downstream applications (Figure 11).

**FIGURE 11:**
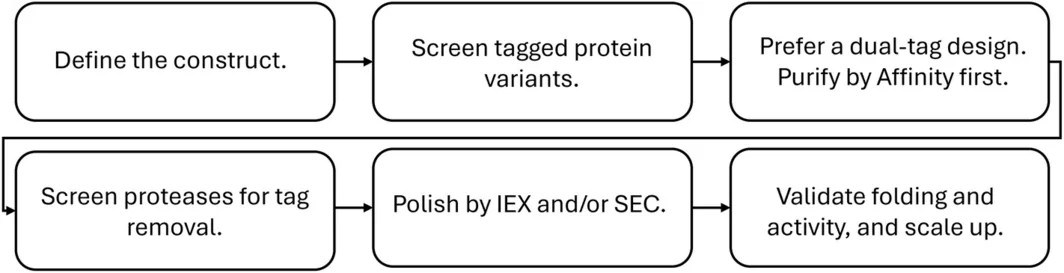
Outline of recommended workflow for purifying a new membrane‐associated receptor domain.

Based on the recommended workflow, our approach can be extended to a wide range of bacterial transmembrane proteins featuring domains exposed to the cell environment, such as efflux pumps, porins, and any other channel‐transporter involved in bacterial vital processes. The features of this workflow that we expect to transfer are: prediction‐guided selection of domain boundaries before cloning; parallel small‐scale screening of fusion partners that differ in size and character, rather than optimization of a single tag; a dual‐tag design that allows capture and polishing to exploit two independent affinity handles and that additionally selects against truncated products lacking the second tag; on‐resin protease cleavage, so that tag removal simultaneously serves as a purification step; and the use of orthogonal biophysical methods to establish not merely that the product is pure, but that it is folded. We expect that the protocols presented here will need to be modified for proteins with vastly different properties: neither of the domains we purified here is strongly hydrophobic, and we would not expect the same approach to necessarily succeed for domains that are extensively membrane‐embedded, or that require the β‐barrel, a partner protein, or a cofactor in order to fold.

## 4 CONCLUSION

Overall, this study establishes a practical and adaptable methodology for producing soluble domains of membrane‐embedded receptors. The combination of solubilization tags, dual‐affinity purification, specific protease cleavage, and comprehensive folding validation provides a reliable workflow that can be readily applied to other TonB‐dependent receptors or challenging bacterial proteins. This approach enables access to high‐quality protein suitable for biophysical and biochemical studies, facilitating future efforts in structural characterization and therapeutic ligand discovery targeting bacterial iron‐acquisition pathways.

## 5 MATERIALS AND METHODS

### 5.1 Cloning of individual domains

The genes encoding for *P. aeruginosa* Secretin/TonB short N‐terminal and N‐terminal plug domains were obtained from the Pseudomonas Genome Database (Winsor et al., 2016). The gene sequences were designed with the *BamHI* restriction site at the 5′ position and with the HexaHistidine tag and the *HindIII* restriction site at the 3′ position. The genes were synthesized from IDT and codon optimized through GeneArt (Thermofisher Scientific) for *E. coli* cells. Both genes were cloned into pBRE vector harboring a HexaHistidine tag; ligation products were transformed into chemically competent Bronze *E. coli* cells via heat‐shock treatment, and the positive clones were selected on 2xYT (Yeast Extract Tryptone) plates containing 100 μg/mL ampicillin. The success of the ligations was tested by enzymatic digestion with *BamHI* and *HindIII* and gel electrophoresis with 1% agarose gel in 1× TAE buffer. The accuracy of the constructs was confirmed by Sanger sequencing from GENEWIZ with primers for T7 promoter and T7 terminator. The alignment of HasR gene sequence with T7 promoter and T7 terminator reads was performed using SnapGene software.

### 5.2 Expression and purification of individual domains

Chemically competent Lucigen C41(DE3) *E. coli* cells were transformed with ligation products obtained from pBRE vector and Secretin/TonB short N‐terminal and N‐terminal plug domains. The transformation mixes were used to inoculate 10 mL 2xYT containing 100 μg/mL ampicillin, then grown overnight at 37°C and 220 rpm. The overnight cultures were used to inoculate the mains cultures with a 1:100 ratio and grown at 37°C and 220 rpm until an OD_600_ of 0.8 was measured. Protein expression was induced by the addition of isopropyl‐b‐D‐1‐thiogalactopyranoside (IPTG) to a final concentration of 1 mM. The mains cultures were then grown overnight at 20°C and 220 rpm. Bacterial pellets from main cultures were recovered by centrifugation (10,000 g, 5 min, 4°C), resuspended in Buffer A (50 mM NaPO_4_ pH 7.5, 150 mM NaCl) supplemented with cOmplete™ Protease Inhibitor Cocktail and DNAase I (1 μg/mL) and lysed by Emulsiflex. Bacterial lysates were centrifugated at 35,000 g, 35 min, 4°C, and the supernatants were recovered for protein purification. N‐terminal plug and Secretin/TonB short N‐terminal domains were purified starting from affinity chromatography with PureCube 100 Compact Cartridge Ni‐INDIGO 1 mL, with a flow rate of 1 mL/min using an AKTA Pure system. After sample application, the column was washed with 30 CV in Buffer A2 (50 mM NaPO_4_ pH 7.5, 500 mM NaCl) supplemented with 20 mM imidazole, and the elution was performed with 40 CV of a linear gradient of the imidazole of the Buffer B from 0% to 100% followed by 10 CV at 100% Buffer B (50 mM NaPO_4_ pH 7.5, 150 mM NaCl, 500 mM imidazole). Protein aliquots corresponding to the elution peak were recovered, assessed using 15% sodium dodecyl sulfate‐polyacrylamide gel electrophoresis (SDS–PAGE), and dialyzed against 1 L Buffer A at 4°C, overnight. After dialysis, SEC with Superdex™ 75 Increase 10/300 GL column was used for protein contaminants coming from the bacterial lysate. The column was equilibrated overnight in Buffer A with 1.2 CV at 0.8 mL/min flow. After sample application, the elution was performed in Buffer A with 1.2 CV at 0.5 mL/min flow. Each protein aliquot corresponding to the elution peak was assessed using 15% SDS–PAGE. N‐terminal plug domain purification was completed by using IEX chromatography with MonoQ column using a gradient of NaCl from 150 mM to 2 M to elute the protein of interest. Protein concentrations were determined by UV adsorption set at 280 nm and adjusted with the predicted molar extinction coefficients calculated by ProtParam tool at ExPASy web portal (ϵ = 12,950 M^−1^ cm^−1^ and ϵ = 2980 M^−1^ cm^−1^, for N‐terminal plug and Secretin/TonB short N‐terminal, respectively).

### 5.3 HasR construct cloning

The gene encoding for *P. aeruginosa* HasR_44‐276_ (including Secretin/TonB short N‐terminal and N‐terminal plug domains) was obtained from the Pseudomonas Genome Database (Winsor et al., 2016). The gene sequence was designed with the *BamHI* restriction site at the 5′ position and with the HexaHistidine tag and the *HindIII* restriction site at the 3′ position (Figure S3a,c). The HasR gene was synthesized from IDT and codon optimized through GeneArt (Thermofisher Scientific) for *E. coli* cells. The gene was cloned into pGST vectors harboring a GST tag sequence and either a thrombin‐ or a TEV‐cleavage site (Figure S3a–c). Similarly, the HasR_44‐276_ gene was amplified from gDNA of *P. aeruginosa PAO1* with the forward primer (5′‐TATA*GGATCC*AGCCAGCAGCAGACCGC‐3′) bringing the *BamHI* restriction site, italicized in the sequence, and the reverse primer (5′‐TAAT*AAGCTT*TTAACCGACCTGCTTGCCCG‐3′) bringing the *HindIII* restriction site, italicized in the sequence. The PCR product was cloned both into pBRE vector harboring a HexaHistidine tag and into pHLT vector harboring a Histidine Lipoyl tag (HLT) (Galloway et al., 2003). All four ligation products were transformed into chemically competent Bronze *E. coli* cells via heat‐shock treatment, and the positive clones were selected on 2xYT plates containing 100 μg/mL ampicillin. The success of the ligation and the accuracy of the constructs were assessed as described above at *Cloning of individual domains*.

### 5.4 HasR construct expression test

Chemically competent Lucigen C41(DE3) *E. coli* cells were transformed with pGST‐thrombin‐HasR‐His6 and pGST‐TEV‐HasR‐His6 constructs via heat shock treatment to produce a GST tag cleavable GST‐HasR‐His6 protein construct (Figure S3a). Similarly, the same *E. coli* cells were transformed with pBRE‐HasR and pHLT‐HasR constructs via heat shock treatment to produce a HexaHistidine tag cleavable His‐HasR and a HLT cleavable HLT‐HasR constructs, respectively. The transformation mix was used to inoculate 10 mL 2xYT containing 100 μg/mL ampicillin, then grown overnight at 37°C and 220 rpm. The overnight culture was used to inoculate the main culture with a 1:100 ratio and grown at 37°C and 220 rpm until an OD_600_ of 0.8 was measured. Protein expression was induced by the addition of IPTG to a final concentration of 1 mM. The main culture was then grown overnight at 20°C and 220 rpm. Bacterial pellet from 1 mL of main culture was recovered by centrifugation (10,000 rpm, 2 min), resuspended in 1 mL of BugBuster® MasterMix (Novagen) and incubated for 30 min at room temperature with shaking. A 20 μL aliquot was saved as total cell protein lysate (TCP). After centrifugation (13,000 rpm, 10 min) the supernatant was recovered as soluble protein fraction (SP); while the pellet was washed in 1 mL of 1:10 diluted BugBuster® MasterMix, dissolved in 300 μL of Solubilization buffer (100 mM Tris–HCl pH 8.2, 8 M urea, 100 mM β‐mercaptoethanol) and centrifugated at 13,000 rpm, 5 min to isolate the insoluble protein fraction (IP). The protein expression was analyzed using 15% SDS–PAGE.

### 5.5 Purification of the HLT‐HasR construct and screening of thrombin cleavage

HLT‐HasR construct was purified by affinity chromatography with HisTrap™ HP 1 mL column at a flow rate of 1 mL/min using an AKTA Pure system. After sample application, the column was washed with 10 CV in Buffer A2, and the elution was performed with 10 CV of a linear gradient of imidazole from Buffer B from 0 to 100%, followed by 5 CV at 100% Buffer B. Protein aliquots corresponding to the elution peak were assessed using 15% SDS–PAGE. The screening of the thrombin cleavage was performed with 4 U of bovine Thrombin (1 U/μL, 7.5 mg/mL) per mg of HLT‐HasR at room temperature with shaking.

### 5.6 HasR construct purification and GST‐tag removal

HasR construct was expressed as described above at *HasR construct expression test*; after centrifugation (10,000*g*, 15 min, 4°C), the bacterial pellet was resuspended in Buffer A supplemented with cOmplete™ Protease Inhibitor Cocktail and DNAase I (1 μg/mL) and lysed by Emulsiflex. Bacterial lysate was centrifugated at 35,000*g*, 35 min, 4°C, and the supernatant was recovered for protein purification (Purification I and Purification II).

#### 5.6.1 Purification I

Purification I was performed as described in Figure 7d. Around 4 g of bacterial pellet were used to isolate HasR construct. The supernatant was incubated with 1 mL (0.5 g) GH‐resin (Cambridge Bioscience LTD) for 2 h, at 4°C with shaking. After centrifugation (500 g, 10 min) GH‐resin was washed with 5 CV three times in Buffer A2 and resuspended in 4 mL of Buffer A. GST tag removal was performed on GH‐resin by bovine thrombin cleavage with 10 U (1 U/μL, 7.5 mg/mL) of the protease at room temperature for 2 h with shaking. The digestion mix including the GH‐resin was transferred to a gravity column and the cleaved HasR construct was eluted by adding 2.5 CV three times of Buffer A. The cleaved product was further purified by affinity chromatography with HisTrap™ excel 1 mL column with a flow rate of 1 mL/min using an AKTA Pure system. After sample application, the column was washed with 20 CV in Buffer A2 and the elution was performed with 40 CV of a linear gradient of the imidazole of the Buffer B from 0% to 100% followed by 10 CV at 100% Buffer B. Protein aliquots corresponding to the elution peak were recovered, assessed using 15% SDS–PAGE, and dialyzed against 1 L Buffer A at 4°C, overnight. HasR construct was separated from digestion byproducts by SEC (Size Exclusion Chromatography). Superdex™ 75 Increase 10/300 GL column was equilibrated overnight in Buffer A with 1.2 CV at 0.8 mL/min flow. After sample application, the elution was performed in Buffer A with 1.2 CV at 0.5 mL/min flow. Each protein aliquot corresponding to the elution peak was assessed using 15% SDS–PAGE. Protein concentration was determined by UV adsorption set at 280 nm and adjusted with the predicted molar extinction coefficient calculated by ProtParam tool at ExPASy web portal (ϵ = 15,930 M^−1^ cm^−1^). Protein purity was calculated from SDS‐PAGE gels, using the GelGenie plugin in QuPath, using the Universal model for segmentation, as previously described (Aquilina et al., 2025).

### 5.7 ESI‐MS

Electrospray ionization mass spectrometry was performed on a Xevo G2 mass spectrometer with data analyzed using MassLynx software (Waters UK) (Yusuf Hamied Department of Chemistry, University of Cambridge, UK).

#### 5.7.1 Purification II

Purification II was performed as described in Figure S5a. Around 140 g of bacterial pellet were used to isolate HasR construct. The supernatant was incubated with 6 × 1 mL GH‐resins (0.5 g) for 2 h, at 4°C with shaking. After centrifugation (500*g*, 10 min) each GH‐resin was washed with 5 CV three times in Buffer A2 and resuspended in 2 mL of Buffer A. GST tag removal was performed on GH‐resin by biotin‐TEV cleavage with 240 U (10 U/μL) of the protease at 4°C, overnight with shaking. The digestion mix including the GH‐resin was transferred to a gravity column and the cleaved HasR construct was eluted by adding 2.5 CV three times of Buffer A. GST tag was eluted from GH‐resin by adding 5 CV three times of Elution Buffer A (50 mM NaPO_4_ pH 8, 150 mM NaCl, 20 mM reduced glutathione). The cleaved product was further purified by affinity chromatography with PureCube 100 Compact Cartridge Ni‐INDIGO 1 mL with a flow rate of 1 mL/min using an AKTA Pure system. After sample application, the column was washed with 30 CV in Buffer A2 supplemented with 20 mM imidazole, and the elution was performed with 40 CV of a linear gradient of the imidazole of the Buffer B from 0% to 100% followed by 10 CV at 100% Buffer B. Protein aliquots corresponding to the elution peak were recovered, assessed using 15% SDS–PAGE, and dialyzed against 1 L Buffer A at 4°C, overnight. Protein concentration was determined by UV adsorption set at 280 nm and adjusted with the predicted molar extinction coefficient calculated by ProtParam tool at ExPASy web portal (ϵ = 15,930 M^−1^ cm^−1^).

### 5.8 Circular dichroism (CD)

CD was measured on the Chirascan CD spectrometer (Applied Photophysics, Leatherhead, UK) in 1‐mm‐pathlength Precision Cells (110‐QS, Hellma Analytics, Müllheim, Germany), using 6 μM of HasR construct, 11 μM of N‐terminal plug, and 17 μM of Secretin/TonB short N‐terminal solutions in Buffer A. The CD spectrum was recorded in the range of 200–250 nm, at 25°C, in three technical replicates (three scans each), and with a bandwidth of 1 nm. Raw CD data were corrected by subtracting the spectrum of the buffer solution. Beta Structure Selection (BeStSel) server was used to determine the protein secondary structure and fold recognition from the CD spectrum, giving an indication of α‐helix and β‐sheets content (Micsonai et al., 2018).

### 5.9 NMR (1D spectrum)

The NMR samples (HasR construct concentration = 155 μM, N‐terminal plug domain concentration = 86 μM and Secretin/TonB domain concentration = 352 μM) were prepared in phosphate buffer (20 mM KPi (Potassium Phosphate) + 150 mM NaCl) with 5% D_2_0. 1D ^1^H spectra were recorded at 25°C, with water suppression using excitation sculpting with gradients (Hwang & Shaka, 1995) on a Bruker Avance III HD 800 MHz spectrometer.

### 5.10 Nano differential scanning fluorimetry (NanoDSF)

NanoDSF was performed with a Prometheus NanoDSF instrument (NanoTemper Technologies) to evaluate the melting temperature of the HasR construct. Protein thermal stability was estimated by calculating the first derivative of the ratio of fluorescence intensity read at 350 nm over 330 nm (dFIR [350 nm/330 nm]) from 20 to 90°C with a 1°C/min rate.

### 5.11 Hydrogen–deuterium exchange mass spectrometry (HDX‐MS)

Hydrogen–deuterium exchange (HDX) is performed as reported in Figures S6 and S7, and using a fully automated, millisecond HDX labeling and online quench‐flow instrument, ms2‐min (Nicoya Lifesciences, CA), connected to an HDX manager (Waters). Samples in the equilibrium buffer are delivered into the labeling mixer and diluted 20‐fold with labeling buffer at 20°C, initiating HDX. The duration of the HDX labeling depends on the mixing loops of varying lengths in the sample chamber of the ms2 min and the velocity of the carrier buffer, calibrated to a precision of 1 ms. The protein is labeled for a range of times from 300 ms to 5 min. Immediately postlabeling, the labeled sample is mixed with quench buffer in a 1:1 ratio at 0°C in the quench mixer to arrest HDX. The sample is then centered on the HPLC injection loop of the ms2 min and sent to the HDX manager. Protein samples are digested onto an enzymatic immobilized pepsin column (Waters) to form peptides. The peptides are trapped on a VanGuard 2.1 mm × 5 mm ACQUITY BEH C18 column (Waters) for 3 min at 175 μL/min and separated on a 1 mm × 100 mm ACQUITY BEH 1.7 μm C18 column (Waters) with a 7 min linear gradient of acetonitrile (5%–40%) supplemented with 0.1% formic acid. The eluted peptides are analyzed on a Synapt G2‐Si mass spectrometer (Waters). Deuterium incorporation into the peptides is measured in DynamX 3.0 (Waters) for peptides identified by separate HDMS^E^ experiments analyzed in ProteinLynx Global Server 3.03 (Waters).

## AUTHOR CONTRIBUTIONS

**Federico Bosetto:** Investigation; writing – original draft; methodology; validation; writing – review and editing; formal analysis. **Maria Zacharopoulou:** Investigation; writing – original draft; methodology; validation; writing – review and editing; formal analysis. **Mark Bycroft:** Investigation; validation. **Monika Kish:** Investigation; validation. **Giovanna Zinzalla:** Investigation; validation. **Pamela J. E. Rowling:** Investigation; validation. **Stephen H. McLaughlin:** Investigation; validation. **Jonathan J. Phillips:** Investigation; validation; supervision; writing – review and editing. **Laura S. Itzhaki:** Writing – review and editing; resources; supervision. **Ioanna Mela:** Conceptualization; funding acquisition; writing – review and editing; project administration; supervision; resources.

## Supporting information


**Figure S1.** Electrospray ionization mass spectrometry of N‐terminal plug, Secretin/TonB short N‐terminal domains and HasR construct. (A) The N‐terminal plug domain showed a major peak at 16.9 kDa, close to the expected size of 15.8 kDa, and a minor peak at 23.6 kDa. (B) The Secretin/TonB short N‐terminal domain size of 13.1 kDa was confirmed. (C) The HasR construct size of 26.4 kDa was also confirmed.
**Figure S2.** 2D NOESY NMR spectra of the N‐terminal plug domain showing long‐range, through‐space contacts that are only present in a folded structure.
**Figure S3.** Illustration about the cloning of HasR‐His construct in pGST vectors, which includes: (A) scheme of the plasmid vectors (pGST‐10 N‐TEV and pGST‐Thrombin), the insert (HasR‐His) and the protein construct (GST‐HasR‐His). (B) Maps of the plasmid vectors (pGST‐10 N‐TEV and pGST‐Thrombin). (C) DNA sequences of plasmid vectors (pGST‐10 N‐TEV and pGST‐Thrombin) and the insert (HasR‐His). HasR‐His construct is cloned in the plasmid vectors by means of *BamHI* and *HindIII*, downstream of the GST, and of the TEV and thrombin cleavage sites.
**Figure S4.** HLT‐HasR construct purification and screening of the thrombin cleavage of the HLT linker. (A) Purification of HLT‐HasR construct by affinity chromatography with HisTrap™ HP 1 mL column. Protein band is denoted by the arrow and protein contaminants at both higher and lower molecular weight are also indicated. (B) Screening of the thrombin cleavage of HLT‐HasR construct at 1–2–4‐6‐8 h and overnight, HLT‐HasR and cleaved HasR protein bands are denoted by arrows, digestion byproducts are also reported.
**Figure S5.** Purification II of GST‐HasR‐His construct. (A) Scheme about the purification process. (B) The protein is first purified by means of GH‐resin, where the GST‐tag is cleaved by TEV, and then (B and C) by affinity chromatography using Ni‐INDIGO column, as reported by the SDS‐PAGE and the chromatogram from the AKTA. HasR construct band is denoted by the arrow, protein contaminants are also indicated. [SP = soluble protein fraction, GH = glutathione].
**Figure S6.** HDX‐MS coverage map of HasR construct. Full sequence coverage (100%) and average 5.02 Redundancy (number of peptides an amino acid appears in) achieved.
**Figure S7.** HDX‐MS uptake plots per identified peptide. Note: analysis software used cannot represent millisecond labeling times, which are necessarily shown on x‐axes as ‘minutes’ instead of ‘milliseconds’.

## Data Availability

The data that support the findings of this study are openly available in Apollo at https://www.repository.cam.ac.uk/home, reference number 1F7A93CE‐6D17‐4B8D‐8639‐D36B521E6CE2.

## References

[pro70825-bib-0001] Abramson J , Adler J , Dunger J , Evans R , Green T , Pritzel A , et al. Accurate structure prediction of biomolecular interactions with AlphaFold 3. Nature. 2024;630(8016):493–500. 10.1038/s41586-024-07487-w PMC11168924 38718835

[pro70825-bib-0002] Alberts B , Johnson A , Lewis J , Raff M , Roberts K , Walter P . Molecular biology of the cell. 4th ed. New York, NY: Garland Publishing Inc; 2002.

[pro70825-bib-0003] Andrews SC , Robinson AK , Rodríguez‐Quiñones F . Bacterial iron homeostasis. Amsterdam, Netherlands: Elsevier; 2003. 10.1016/S0168-6445(03)00055-X 12829269

[pro70825-bib-0004] Aquilina M , Wu NJW , Kwan K , Bušić F , Dodd J , Nicolás‐Sáenz L , et al. GelGenie: an AI‐powered framework for gel electrophoresis image analysis. Nat Commun. 2025;16(1):4087. 10.1038/s41467-025-59189-0 PMC12053679 40324991

[pro70825-bib-0005] Becker KW , Skaar EP . Metal limitation and toxicity at the interface between host and pathogen. FEMS Microbiol Rev. 2014;38:1235–1249. 10.1111/1574-6976.12087 PMC4227937 25211180

[pro70825-bib-0006] Blum M , Andreeva A , Florentino LC , Chuguransky SR , Grego T , Hobbs E , et al. InterPro: the protein sequence classification resource in 2025. Nucleic Acids Res. 2025;53(D1):D444–D456. 10.1093/nar/gkae1082 PMC11701551 39565202

[pro70825-bib-0007] Carpenter EP , Beis K , Cameron AD , Iwata S . Overcoming the challenges of membrane protein crystallography. Curr Opin Struct Biol. 2008;18(5):581–586. 10.1016/j.sbi.2008.07.001 PMC2580798 18674618

[pro70825-bib-0008] Cavanagh J , Fairbrother WJ , Palmer AG III , Rance M , Skelton NJ . Protein NMR spectroscopy. 2nd ed. Burlington, MA: Elsevier; 2007. 10.1016/B978-0-12-164491-8.X5000-3

[pro70825-bib-0009] de Marco A . Recent advances in recombinant production of soluble proteins in *E. coli* . Microb Cell Fact. 2025;24(1):21. 10.1186/s12934-025-02646-8 PMC11736966 39815265

[pro70825-bib-0010] de Marco A , Deuerling E , Mogk A , Tomoyasu T , Bukau B . Chaperone‐based procedure to increase yields of soluble recombinant proteins produced in *E. coli* . BMC Biotechnol. 2007;7:32. 10.1186/1472-6750-7-32 PMC1904446 17565681

[pro70825-bib-0011] Dent AT , Wilks A . Contributions of the heme coordinating ligands of the *Pseudomonas aeruginosa* outer membrane receptor HasR to extracellular heme sensing and transport. J Biol Chem. 2020;295(30):10456–10467. 10.1074/jbc.ra120.014081 PMC7383377 32522817

[pro70825-bib-0012] Dyson MR , Shadbolt SP , Vincent KJ , Perera RL , McCafferty J . Production of soluble mammalian proteins in *Escherichia coli*: identification of protein features that correlate with successful expression. BMC Biotechnol. 2004;4:32. 10.1186/1472-6750-4-32 PMC544853 15598350

[pro70825-bib-0013] Elfadadny A , Ragab RF , AlHarbi M , Badshah F , Ibáñez‐Arancibia E , Farag A , et al. Antimicrobial resistance of *Pseudomonas aeruginosa*: navigating clinical impacts, current resistance trends, and innovations in breaking therapies. Front Microbiol. 2024;15:1374466. 10.3389/fmicb.2024.1374466 PMC11026690 38646632

[pro70825-bib-0014] Francis DM , Page R . Strategies to optimize protein expression in *E. coli* . Curr Protoc Protein Sci. 2010;5(1):5.24.1–5.24.29. 10.1002/0471140864.ps0524s61 PMC7162232 20814932

[pro70825-bib-0015] Galloway CA , Sowden MP , Smith HC . Increasing the yield of soluble recombinant protein expressed in *E. coli* by induction during late log phase. Biotechniques. 2003;34(3):524–530. 10.2144/03343st04 12661158

[pro70825-bib-0016] Gao K , Oerlemans R , Groves MR . Theory and applications of differential scanning fluorimetry in early‐stage drug discovery. Biophys Rev. 2020;12(1):85–104. 10.1007/s12551-020-00619-2 PMC7040159 32006251

[pro70825-bib-0017] Greenfield NJ . Using circular dichroism spectra to estimate protein secondary structure. Nat Protoc. 2006;1(6):2876–2890. 10.1038/nprot.2006.202 PMC2728378 17406547

[pro70825-bib-0018] Hardy D , Bill RM , Jawhari A , Rothnie AJ . Overcoming bottlenecks in the membrane protein structural biology pipeline. Biochem Soc Trans. 2016;44(3):838–844. 10.1042/BST20160049 27284049

[pro70825-bib-0019] Hoffmann A , Grubwieser P , Bumann D , Haschka D , Weiss G . Tackling microbial iron homeostasis: novel antimicrobial strategies. Trends Pharmacol Sci. 2025;46(10):1004–1017. 10.1016/j.tips.2025.08.003 40914687

[pro70825-bib-0020] Hong P , Koza S , Bouvier ESP . A review size‐exclusion chromatography for the analysis of protein biotherapeutics and their aggregates. J Liq Chromatogr Relat Technol. 2012;35:2923–2950. 10.1080/10826076.2012.743724 PMC3556795 23378719

[pro70825-bib-0021] Hwang TL , Shaka AJ . Water suppression that works. Excitation sculpting using arbitrary wave‐forms and pulsed‐field gradients. J Magn Reson A. 1995;112(2):275–279. 10.1006/jmra.1995.1047

[pro70825-bib-0022] Jia B , Jeon CO . High‐throughput recombinant protein expression in *Escherichia coli*: current status and future perspectives. Open Biol. 2016;6(8):160196. 10.1098/rsob.160196 PMC5008019 27581654

[pro70825-bib-0023] Jumper J , Evans R , Pritzel A , Green T , Figurnov M , Ronneberger O , et al. Highly accurate protein structure prediction with AlphaFold. Nature. 2021;596(7873):583–589. 10.1038/s41586-021-03819-2 PMC8371605 34265844

[pro70825-bib-0024] Konermann L , Pan J , Liu Y‐H . Hydrogen exchange mass spectrometry for studying protein structure and dynamics. Chem Soc Rev. 2011;40(3):1224–1234. 10.1039/C0CS00113A 21173980

[pro70825-bib-0025] Krieg S , Huché F , Diederichs K , Izadi‐Pruneyre N , Lecroisey A , Wandersman C , et al. Heme uptake across the outer membrane as revealed by crystal structures of the receptor–hemophore complex. Proc Natl Acad Sci. 2009;106(4):1045–1050. 10.1073/pnas.0809406106 PMC2633585 19144921

[pro70825-bib-0026] Krishnarjuna B , Ramamoorthy A . Detergent‐free isolation of membrane proteins and strategies to study them in a near‐native membrane environment. Biomolecules. 2022;12(8):1076. 10.3390/biom12081076 PMC9406181 36008970

[pro70825-bib-0027] Krūmiņa A , Zeltiņa I , Simsone P , Eulitz E , Reinis A , Vīksna L . Mechanisms of *Pseudomonas aeruginosa* resilience against antibiotic treatment and outlooks of emerging treatment strategies. Medicina (Kaunas). 2026;62:163. 10.3390/medicina62010163 PMC12843202 41597449

[pro70825-bib-0028] Lebendiker M , Danieli T . Production of prone‐to‐aggregate proteins. FEBS Lett. 2014;588(2):236–246. 10.1016/j.febslet.2013.10.044 24211444

[pro70825-bib-0029] Lister PD , Wolter DJ , Hanson ND . Antibacterial‐resistant *Pseudomonas aeruginosa*: clinical impact and complex regulation of chromosomally encoded resistance mechanisms. Clin Microbiol Rev. 2009;22(4):582–610. 10.1128/CMR.00040-09 PMC2772362 19822890

[pro70825-bib-0030] Llamas MA , Sánchez‐Jiménez A . Iron homeostasis in *Pseudomonas aeruginosa*: targeting iron acquisition and storage as an antimicrobial strategy. Adv Exp Med Biol. 2022;1386:29–68. 10.1007/978-3-031-08491-1_2 36258068

[pro70825-bib-0031] Malki I , Simenel C , Wojtowicz H , Cardoso de Amorim G , Prochnicka‐Chalufour A , Hoos S , et al. Interaction of a partially disordered Antisigma factor with its partner, the signaling domain of the TonB‐dependent transporter HasR. PLoS One. 2014;9(4):e89502. 10.1371/journal.pone.0089502 PMC3984077 24727671

[pro70825-bib-0032] Marchetti M , de Bei O , Bettati S , Campanini B , Kovachka S , Gianquinto E , et al. Iron metabolism at the interface between host and pathogen: from nutritional immunity to antibacterial development. Int J Mol Sci. 2020;21:2145. 10.3390/ijms21062145 PMC7139808 32245010

[pro70825-bib-0033] Masson GR , Burke JE , Ahn NG , Anand GS , Borchers C , Brier S , et al. Recommendations for performing, interpreting and reporting hydrogen deuterium exchange mass spectrometry (HDX‐MS) experiments. Nat Methods. 2019;16(7):595–602. 10.1038/s41592-019-0459-y PMC6614034 31249422

[pro70825-bib-0034] Meng EC , Goddard TD , Pettersen EF , Couch GS , Pearson ZJ , Morris JH , et al. UCSF ChimeraX: tools for structure building and analysis. Protein Sci. 2023;32(11):1–13. 10.1002/pro.4792 PMC10588335 37774136

[pro70825-bib-0035] Micsonai A , Wien F , Bulyáki É , Kun J , Moussong É , Lee YH , et al. BeStSel: a web server for accurate protein secondary structure prediction and fold recognition from the circular dichroism spectra. Nucleic Acids Res. 2018;46(W1):W315–W322. 10.1093/nar/gky497 PMC6031044 29893907

[pro70825-bib-0036] Miller WR , Arias CA . ESKAPE pathogens: antimicrobial resistance, epidemiology, clinical impact and therapeutics. Nat Rev Microbiol. 2024;22:598–616. 10.1038/s41579-024-01054-w PMC13147291 38831030

[pro70825-bib-0037] Ochsner UA , Johnson Z , Vasil ML . Genetics and regulation of two distinct haem‐uptake systems, phu and has, in *Pseudomonas aeruginosa* . Microbiology (Reading). 2000;146(Pt 1):185–198. 10.1099/00221287-146-1-185 10658665

[pro70825-bib-0038] Otero‐Asman JR , García‐García AI , Civantos C , Quesada JM , Llamas MA . *Pseudomonas aeruginosa* possesses three distinct systems for sensing and using the host molecule haem. Environ Microbiol. 2019;21(12):4629–4647. 10.1111/1462-2920.14773 31390127

[pro70825-bib-0039] Pang Z , Raudonis R , Glick BR , Lin TJ , Cheng Z . Antibiotic resistance in *Pseudomonas aeruginosa*: mechanisms and alternative therapeutic strategies. Biotechnol Adv. 2019;37:177–192. 10.1016/j.biotechadv.2018.11.013 30500353

[pro70825-bib-0040] Pucci F , Rooman M . Improved insights into protein thermal stability: from the molecular to the structurome scale. Philos Trans R Soc A Math Phys Eng Sci. 2016;374(2080):20160141. 10.1098/rsta.2016.0141 PMC5052726 27698032

[pro70825-bib-0041] Rehm T , Huber R , Holak TA . Application of NMR in structural proteomics. Structure. 2002;10(12):1613–1618. 10.1016/S0969-2126(02)00894-8 12467568

[pro70825-bib-0042] Sánchez‐Jiménez A , Marcos‐Torres FJ , Llamas MA . Mechanisms of iron homeostasis in *Pseudomonas aeruginosa* and emerging therapeutics directed to disrupt this vital process. J Microbial Biotechnol. 2023;16:1475–1491. 10.1111/1751-7915.14241 PMC10281387 36857468

[pro70825-bib-0043] Schrödinger L . The PyMOL molecular graphics system, version 2.5. New York: Schrödinger, LLC; 2021.

[pro70825-bib-0044] Smith AD , Wilks A . Differential contributions of the outer membrane receptors PhuR and HasR to heme acquisition in *Pseudomonas aeruginosa* . J Biol Chem. 2015;290(12):7756–7766. 10.1074/jbc.M114.633495 PMC4367277 25616666

[pro70825-bib-0045] Sørensen HP , Mortensen KK . Advanced genetic strategies for recombinant protein expression in *Escherichia coli* . J Biotechnol. 2005;115(2):113–128. 10.1016/j.jbiotec.2004.08.004 15607230

[pro70825-bib-0046] Studier FW . Protein production by auto‐induction in high‐density shaking cultures. Protein Expr Purif. 2005;41(1):207–234. 10.1016/j.pep.2005.01.016 15915565

[pro70825-bib-0047] Sun C , Liang J , Shi R , Gao X , Zhang R , Hong F , et al. Tobacco etch virus protease retains its activity in various buffers and in the presence of diverse additives. Protein Expr Purif. 2012;82(1):226–231. 10.1016/j.pep.2012.01.005 22285121

[pro70825-bib-0048] Tungekar AA , Castillo‐Corujo A , Ruddock LW . So you want to express your protein in *Escherichia coli*? Essays Biochem. 2021;65(2):247–260. 10.1042/EBC20200170 33955451

[pro70825-bib-0049] Winsor GL , Griffiths EJ , Lo R , Dhillon BK , Shay JA , Brinkman FSL . Enhanced annotations and features for comparing thousands of Pseudomonasgenomes in the Pseudomonas genome database. Nucleic Acids Res. 2016;44(D1):D646–D653. 10.1093/nar/gkv1227 PMC4702867 26578582

