# Supplementary material for: A generalizable method for the purification and biophysical characterization of bacterial membrane receptors
Source: Protein Sci. 2026 Oct 15;35(11):e70825. doi: 10.1002/pro.70825 (PMC13670019; doi:10.1002/pro.70825)
Supplement: Supplementary file 1 [file PRO-35-e70825-s001.docx]

**A generalisable method for the purification and biophysical characterisation of bacterial membrane receptors**

Federico Bosetto*^1^, Maria Zacharopoulou*^1^, Mark Bycroft^1^, Monika Kish^2,3^, Giovanna Zinzalla^1^, Pamela J. E. Rowling^1^, Stephen H. McLaughlin^4^, Jonathan J. Phillips^2,3^, Laura S. Itzhaki^1^, Ioanna Mela^1^

^1^Department of Pharmacology, University of Cambridge, Tennis Court Road, Cambridge, CB2 1PD, UK

^2^Living Systems Institute, University of Exeter, Exeter, EX4 4QD, UK

^3^Department of Biosciences, University of Exeter, Exeter, EX4 4QD, UK

^4^Medical Research Council Laboratory of Molecular Biology, Francis Crick Avenue, Cambridge Biomedical Campus, Cambridge, CB2 0QH, UK

*These authors have contributed equally to this work.

Corresponding author: Ioanna Mela, im337@cam.ac.uk

**Keywords:** HasR receptor, protein purification, NMR, HDX-MS, biophysical characterisation

**Supplementary Materials**

**Supplementary Figure S1.** Electrospray ionisation mass spectrometry of N-terminal plug, Secretin/TonB short N-terminal domains and HasR construct. **(A)** The N-terminal plug domain showed a major peak at 16.9 kDa, close to the expected size of 15.8 kDa, and a minor peak at 23.6 kDa. **(B)** The Secretin/TonB short N-terminal domain size of 13.1 kDa was confirmed. **(C)** The HasR construct size of 26.4 kDa was also confirmed.

**Supplementary Figure S2:** 2D NOESY NMR spectra of the N-terminal plug domain showing long-range, through-space contacts that are only present in a folded structure.

**Supplementary Figure S3.** Illustration about the cloning of HasR-His construct in pGST vectors, which includes: **(A)** scheme of the plasmid vectors (pGST-10N-TEV and pGST-Thrombin), the insert (HasR-His) and the protein construct (GST-HasR-His). **(B)** Maps of the plasmid vectors (pGST-10N-TEV and pGST-Thrombin). **(C)** DNA sequences of plasmid vectors (pGST-10N-TEV and pGST-Thrombin) and the insert (HasR-His). HasR-His construct is cloned in the plasmid vectors by means of *BamHI* and *HindIII,* downstream of the GST, and of the TEV and thrombin cleavage sites.

**Supplementary Figure S4.** HLT-HasR construct purification and screening of the thrombin cleavage of the HLT linker. **(A)** Purification of HLT-HasR construct by affinity chromatography with HisTrap^TM^ HP 1 ml column. Protein band is denoted by the arrow and protein contaminants at both higher and lower molecular weight are also indicated. **(B)** Screening of the thrombin cleavage of HLT-HasR construct at 1-2-4-6-8 hours and overnight, HLT-HasR and cleaved HasR protein bands are denoted by arrows, digestion byproducts are also reported.

**Supplementary Figure S5**. Purification II of GST-HasR-His construct. **(A)** Scheme about the purification process. **(B)** The protein is first purified by means of GH-resin, where the GST-tag is cleaved by TEV, and then **(B and C)** by affinity chromatography using Ni-INDIGO column, as reported by the SDS-PAGE and the chromatogram from the AKTA. HasR construct band is denoted by the arrow, protein contaminants are also indicated. [SP= soluble protein fraction, GH= glutathione].

**Supplementary Figure S6.** HDX-MS coverage map of HasR construct. Full sequence coverage (100%) and average 5.02 Redundancy (number of peptides an amino acid appears in) achieved.

**Supplementary Figure S7.** HDX-MS uptake plots per identified peptide. Note: analysis software used cannot represent millisecond labelling times, which are necessarily shown on x-axes as ‘minutes’ instead of ‘milliseconds’.
